# A novel multiplexed immunoassay identifies CEA, IL-8 and prolactin as prospective markers for Dukes’ stages A-D colorectal cancers

**DOI:** 10.1186/s12014-015-9081-x

**Published:** 2015-04-08

**Authors:** Sadia Mahboob, Seong Beom Ahn, Harish R Cheruku, David Cantor, Emma Rennel, Simon Fredriksson, Gabriella Edfeldt, Edmond J Breen, Alamgir Khan, Abidali Mohamedali, Md Golam Muktadir, Shoba Ranganathan, Sock-Hwee Tan, Edouard Nice, Mark S Baker

**Affiliations:** Australian School of Advanced Medicine, Faculty of Medicine and Human Sciences, Macquarie University, Rm1, Level 1, 75 Talavera Road, Sydney, NSW 2109 Australia; Department of Chemistry and Biomolecular Sciences, Faculty of Science, Macquarie University, Sydney, NSW 2109 Australia; Olink Bioscience, Dag Hammarskjölds Väg, 54A, 75183 Uppsala, Sweden; Australian Proteome Analysis Facility, Macquarie University, Sydney, NSW 2109 Australia; School of Science and Health, University of Western Sydney, NSW, Australia; Department of Biochemistry and Molecular Biology, Monash University, Clayton Campus, Melbourne, VIC 3800 Australia

**Keywords:** Multiplex immunoassay, Plasma biomarker, Colorectal cancer

## Abstract

**Background:**

Current methods widely deployed for colorectal cancers (CRC) screening lack the necessary sensitivity and specificity required for population-based early disease detection. Cancer-specific protein biomarkers are thought to be produced either by the tumor itself or other tissues in response to the presence of cancers or associated conditions. Equally, known examples of cancer protein biomarkers (e.g., PSA, CA125, CA19-9, CEA, AFP) are frequently found in plasma at very low concentration (pg/mL-ng/mL). New sensitive and specific assays are therefore urgently required to detect the disease at an early stage when prognosis is good following surgical resection. This study was designed to meet the longstanding unmet clinical need for earlier CRC detection by measuring plasma candidate biomarkers of cancer onset and progression in a clinical stage-specific manner. EDTA plasma samples (1 μL) obtained from 75 patients with Dukes’ staged CRC or unaffected controls (age and sex matched with stringent inclusion/exclusion criteria) were assayed for expression of 92 human proteins employing the Proseek® Multiplex Oncology I proximity extension assay. An identical set of plasma samples were analyzed utilizing the Bio-Plex Pro™ human cytokine 27-plex immunoassay.

**Results:**

Similar quantitative expression patterns for 13 plasma antigens common to both platforms endorsed the potential efficacy of Proseek as an immune-based multiplex assay for proteomic biomarker research. Proseek found that expression of Carcinoembryonic Antigen (CEA), IL-8 and prolactin are significantly correlated with CRC stage.

**Conclusions:**

CEA, IL-8 and prolactin expression were found to identify between control (unaffected), non-malignant (Dukes’ A + B) and malignant (Dukes’ C + D) stages.

**Electronic supplementary material:**

The online version of this article (doi:10.1186/s12014-015-9081-x) contains supplementary material, which is available to authorized users.

## Background

CRC is the third most commonly diagnosed cancer worldwide with over 694,000 deaths (8.5% of all cancer deaths) in 2012, with Australia and New Zealand having the highest incidence rates (44.8 and 32.2 per 100,000 in men and women respectively) [1].

Various staging systems have been developed to describe the progression of the disease based on the size, location and spread of the tumour to distant organs (e.g., Tumour-Node-Metastasis (TNM) staging systems, Australian Clinico-pathological staging (ACPS) system and Dukes’ staging system [2,3]). Patient prognosis inversely correlates with tumour stage at the time of diagnosis [4,5]. Once metastases becomes clinically observable, prognosis is extremely poor with survival often measured in months [6]. Currently, we are unable to detect patients with clinically silent metastases, possibly linked to poor outcome. Despite the availability of numerous screening strategies, aggressive surgical therapy and extensive research on the molecular basis of CRC, early detection of the disease remains problematic. Population-based CRC screening programmes can reduce morbidity and mortality through the early identification of surgically-treatable disease. However, there is currently a gap in translational research between identification of potential new biomarkers and development of Food and Drug Association (FDA) approved diagnostic tests [7]. Most diagnostic tests available to date are based on a single protein biomarker [8]. This concept is hazardous in the clinical setting as biological systems are interdependent and highly complex with inherent false positives subject to the genomic instability of cancers [9]. It is now widely accepted that panels of biomarkers will be required to achieve the increased sensitivity and specificity necessary for population-based screening [10]. The use of a pan-cellular field such as proteomics could help identify protein expression profiles associated with CRC progression that may prove to be more reliable than single biomarker based assessment.

Simultaneous assessment using a multiple biomarker strategy necessitates the development of multiplex high-throughput technologies with sufficient sensitivity and specificity to detect CRC early [9]. Multiplexing or simultaneous quantitation of several biomarkers in plasma can indicate the protein expression profiles involved in tumour formation, progression and metastasis. Under carefully controlled experimental conditions, multiplexed assays can identify many (96) low abundance candidate proteins using minimal sample volumes (1 μl) [11]. An example of this technology is the proximity extension assay (PEA) which has recently been developed by Olink Biosciences from Uppsala, Sweden [12].

This study was designed to meet a longstanding clinical need for earlier CRC detection by identifying plasma biomarkers of CRC onset and progression using the Proseek® Multiplex Oncology kit I (Proseek assay). In detail, Proseek assay employs PEA technology to quantitate 92 potential oncoproteins using only 1 μL of human plasma [12], where samples are treated with matched antibody pairs that are tagged with DNA reporter molecules. Once the antibodies are bound to their respective antigen the corresponding DNA tails form an amplicon that can be quantified by high-throughput real time PCR which generates a measurable fluorescent signal that directly correlates with abundance [13]. This PEA-based approach provides a platform for accurate quantification of multiple (96) low abundance oncoproteins from biological samples. Here, we aimed to validate PEA results with an existing benchmark multiplexed technology, namely the Bio-Plex Pro™ human cytokine 27-plex kit [14] (Bio-Plex), which is a bead-based multiplex immunoassay, measures the concentrations of 27 cytokines, chemokines or growth factors.

## Results

### Proseek® multiplex oncology I assay

The expression levels of 92 potential protein biomarkers (Additional file 1: Table S3) in each of the 75 plasma samples from CRC patients and healthy controls were evaluated simultaneously using the Proseek assay. The levels of 8 oncoproteins (CEA, IL-8, prolactin, amphiregulin, PDGF-BB, IL-6, CXCL11 and CXCL5) differed significantly between various individual CRC stages (Table 1).

**Table 1:** **Tukey-honest significant differences post-hoc test for Proseek data [Stage specific (A-D)] and healthy unaffected control (group E)**

**Candidate Biomarker**	**Comparison**	**Up/Down of expression**	**Adjusted p-value**	**Previous studies referring CRC associations**
**CEA**	D/A	↑	0	[15-21]
	D/E	↑	1.70E-12	
	D/B	↑	4.13E-12	
	D/C	↑	0.0001	
	C/A	↑	0.0076	
	C/E	↑	0.0304	
**IL-8**	D/E	↑	1.23E-06	[44,47]
	D/A	↑	2.96E-05	
**Prolactin**	C/D	↑	1.24E-05	[49-51]
	C/E	↑	2.89E-05	
**Amphiregulin**	D/A	↑	8.95E-07	[54]
	D/C	↑	1.46E-06	
**PDGF-BB**	D/A	↑	3.02E-05	[22,55]
**IL-6**	B/A	↑	0.0024	[43,56]
	B/E	↑	0.0124	
**CXCL11**	D/C	↑	0.0155	[57]
	D/A	↑	0.0387	
**CXCL5**	D/A	↑	0.0424	[58-61]

Additional file 1: Tables S3 and S4 list the complete statistical analyses on this data. Twelve (12) of the target biomarkers were found to have expression levels below the Proseek LOD.

Specifically, CEA was found to be the overexpressed protein measured in stage D when compared with any other CRC stage (i.e., either Dukes’ A, B or C) and/or healthy unaffected controls (for all stage D comparisons *P*s were ≤ 0.0001). In addition, CEA was also overexpressed in stage C when compared with stage A (*P* = 0.0076) and/or healthy controls (*P* = 0.0304). Previous studies have also shown elevated CEA expression in Dukes’ stage C and D CRC [15-21].

**Figure 1:**
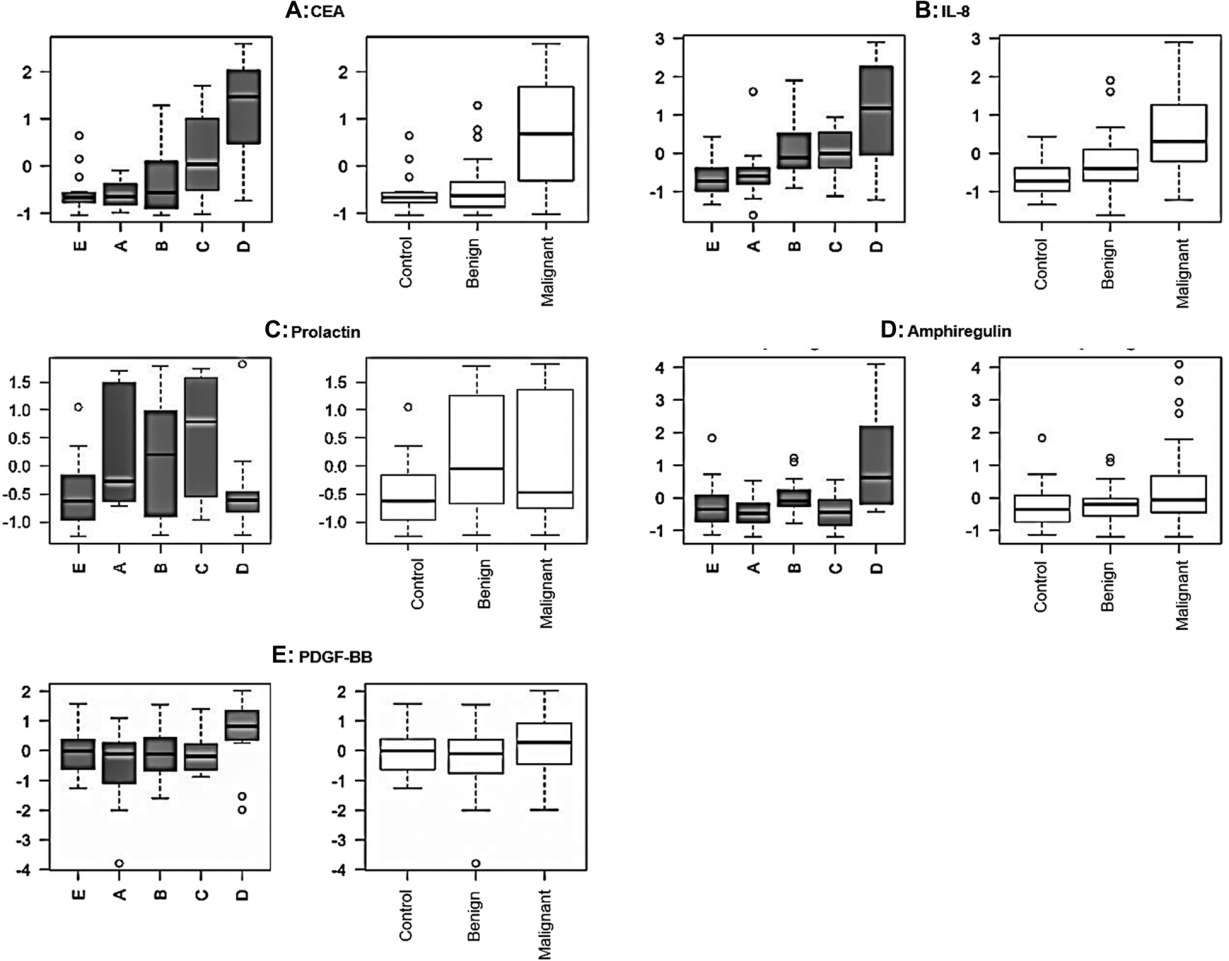
**Panels (P) (A, B, C, D & E) representing protein abundance data for CEA (A), IL-8 (B) prolactin (C), amphiregulin (D) and PDGF-BB (E) in individual Dukes’ stage A, B, C & D with E controls; pooled group (defined control, non-malignant, malignant groups).** Values were determined by Proseek PEA using CRC patient EDTA plasmas and was based on z-scores (log2 scale).

Differences in IL-8 expression were observed in stage A to D comparisons (*P* = 2.96E-05) and stage D to healthy control comparisons (*P* = 1.23E-06). Interestingly, levels of prolactin were elevated in Dukes’ stage C compared with stage D (*P* = 1.24E-05) and healthy controls (*P* = 2.89E-05). Prolactin levels were found to consistently increase as disease progressed from controls through CRC Dukes’ stages A-C (Figure 1, P5). Amphiregulin was overexpressed in stage D when compared with stages A (*P* = 8.96E-07) and C (*P* = 1.46E-06), while PDGF-BB was elevated in stage D compared with stage A (*P* = 3.02E-05). It was also noted that IL-6 showed a higher expression in stage B when compared to stage A and healthy unaffected controls (*P* ≤ 0.0024). Chemokine (C-X-C motif) ligand CXCL11 and CXCL5 had a higher expression at stage D when compared with stage A (*P* = 0.0155). It was interesting to note that some of the previously reported biomarker oncoproteins (e.g., IL-4, CAIX, TNF-α, MCP-1, GM-CSF, VEGF, TIE2, IL17, IL-6, IFNG) did not display differential expression between Dukes’ CRC stages (*P* ≈ 1.0) [22-32].

To determine whether changes were observed when CRC data were pooled into control (group E), non-malignant (stages A + B combined) or malignant groups (stages C + D combined), a Tukey honest significant differences post-hoc ANOVA (Type II) test was performed and Q values calculated, where Q values are a measure of statistical significance in terms of false discovery rate (Table 2).

**Table 2:** **Q values of significantly altered potential biomarker proteins between pooled CRC groups**

**Candidate Biomarker**	**Comparison**	**Difference**	**Lower CI**	**Upper CI**	**Q-value**
CEA	Malignant/Non-malignant	1.97	1.08	2.85	0
CEA	Malignant/Healthy	2.11	1.02	3.19	0
IL.8	Malignant/Healthy	1.22	0.14	2.31	0
Prolactin	Non-malignant/Healthy	1.1	0.02	2.19	0.04

This study showed expression of three biomarker oncoproteins (CEA, IL-8 and prolactin) were altered when pooled CRC groups (i.e., control, non-malignant, malignant) were considered.

Comparison between pooled controls, non-malignant and malignant groups with individually staged patients indicated CEA and IL-8 were both considerably upregulated in malignant compared to healthy controls. Additionally, as expected [20] CEA was overexpressed in comparisons between non-malignant and malignant groups (Q-value = 0). In contrast, prolactin demonstrated a noticeable Dukes’ stage-dependant increase in expression until metastasis occurred beyond lymph nodes (i.e., is elevated up to stage C). Once metastasized to distal organs (Dukes’ stage D), plasma prolactin expression levels returned to approximately normal control (group E) levels. Additionally, an increase was found between control and non-malignant pooled groups for prolactin values.

**Figure 2:**
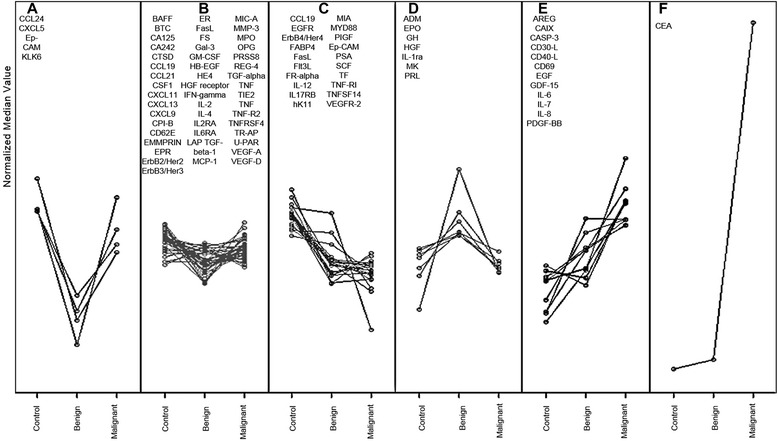
**SOMs trends (A-F) of control, non-malignant and malignant CRC groups normalised median Proseek biomarker protein expression scores, showing these can be clustered into six distinct trends.**

The Proseek multiplexed assay data strongly suggests the combined use of CEA, IL-8 and prolactin expression as potential combined diagnostic indicators of Dukes’ stage with their abundance positively correlating with metastatic progression. However, as target proteins display differences in expression trends, SOMs were used to cluster and visualise pooled data into one of six discernible expression trends (Figure 2).

SOMs assembled the data into the six trends (Figure 2), where median values were used as they are less susceptible to variation [33]. The largest differences were observed between either the non-malignant or the malignant patient groups against control patients’ plasmas. SOM trend A biomarkers decrease between controls and non-malignant plasmas but increase again to similar levels when malignant plasmas are compared to controls. All trend B biomarkers show no major change irrespective of stage. In an opposite manner to trend A, trend D biomarkers increase in non-malignant but decrease again in malignancy. Trend C biomarkers steadily decreased biomarker expression from healthy to malignant and may be useful to distinguish healthy patients from malignant cancers. A converse trend pattern was observed for both trends E (amphiregulin, CA19, caspase 3, CD30, CD40, CD69, EGF, GDF15, Il-6, Il-7, Il-8, PDGF-BB) and F (CEA) with strong steady increases observed during progression. Data shown in trend F demonstrates the power CEA has over all other protein biomarkers examined in the Proseek panel for distinguishing malignant from either non-malignant CRC and/or healthy patients.

### Bio-Plex Pro™ human cytokine 27-plex immunoassay

Where possible the significant differences observed using the Proseek assay were reproduced/validated using an established antibody-based multiplexed detection system, namely the Bio-plex Pro™ human cytokine 27-plex immunoassay. The Dukes’ CRC stage specific analyses identified 6 target proteins (IFN-G, IL-4, IL-8, MCP-1, MIP-1 and PDGF-BB) that were each significantly elevated in stage D plasmas compared with healthy unaffected controls (*P* < 0.05) (Table 3). Additional file 1: Tables S5 and S6 summarize the statistical analyses conducted on the Bio-plex data.

**Table 3:** **Tukey-honest significant differences post-hoc test for Bio-Plex data [Stage specific (A-D)] and healthy unaffected control (group E)**

**Candidate Biomarker**	**Comparison**	**Adjusted p-value**	**Previous studies referencing CRC association**
IL-8	D/E	6.00E-05	[44,47]
	A/E	6.00E-03	
	B/E	2.00E-03	
PDGF-BB	D/E	2.00E-07	[22,55]
	D/A	4.00E-03	
CCL2	D/E	5.00E-04	[61-63]
IFNG	D/E	0.002	[63]
IL-4	D/E	0.004	[64]
CCL3	D/E	0.004	[65]

In detail, significant differences in IL-8 expression were observed in stage A, B and D when compared to healthy controls (*P* = 6.00E-05, 6.00E-03 and 2.00E-03 respectively). Additionally, PDGF-BB was significantly elevated in stage D compared with healthy controls or stage A (*P*s = 2.00E-07 and 4.00E-03 respectively). It was also noted that monocyte chemotactic protein-1/C-C motif chemokine 2 (CCL2) showed a significantly higher expression in stage D when compared to healthy unaffected controls (*P* = 5.00E-04). Furthermore, IFNG, IL-4 and monocyte chemotactic protein-1/C-C motif chemokine 3 (CCL3) also exhibited significant overexpression in stage D when compared to healthy controls (*P* ≤ 0.05).

**Figure 3:**
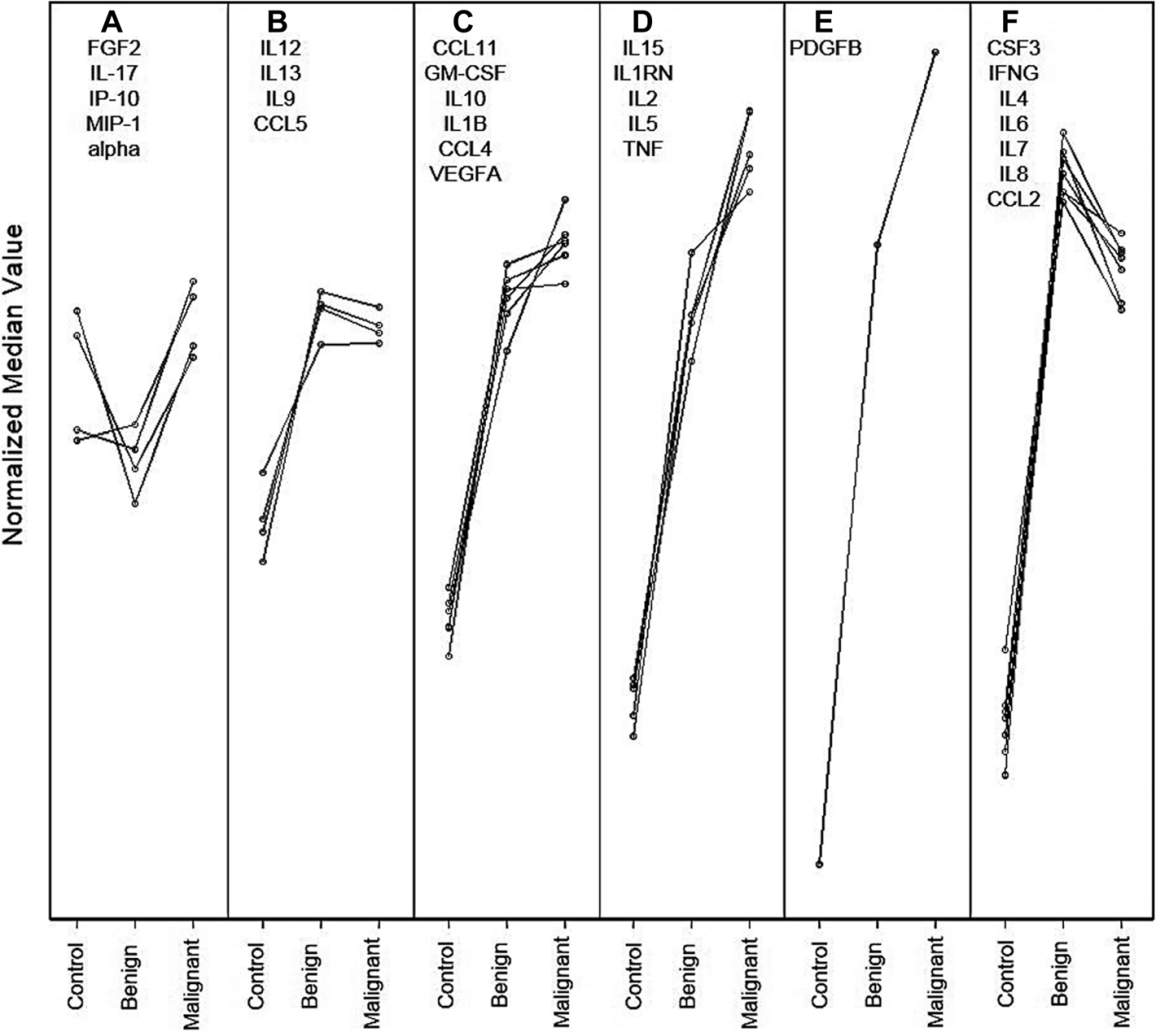
**SOMs trends (A-F) of control, non-malignant and malignant CRC groups normalised median Bio-Plex biomarker protein expression scores showing these can clustered into six distinct patterns/trends.**

Comparison between pooled control, non-malignant and malignant groups with individually staged patients indicated that four proteins (i.e., G-CSF, IL-4, IL-8 and MCP-1) were more highly expressed between non-malignant and healthy cohorts whilst nine proteins (G-CSF, IFN-G, IL-4, IL-6, IL-8, IL-9, MCP-1, MIP-1B and PDGF-BB) were higher in the metastatic group compared to healthy cohorts (Additional file 1: Table S6). SOM analyses of the same data was performed (Figure 3).

Analysis of the SOM data highlights six different trends (A-F) of patient plasma cytokine response to CRC progression. Trend A shows a variable response, whilst trend B shows an increase in cytokine/chemokine expression in both non-malignant and malignant CRC groups above healthy controls. Trend C also displays increased expression in both cancer groups compared to healthy controls with additional slight increases in malignant above non-malignant groups. The tendency for increased expression as cancers progress was more pronounced in trends D and E, whilst in trend F the increase in non-malignant over control groups was followed by a small decrease when the malignant group was compared to the non-malignant group. Collectively, these observations may hold some prognostic value for evaluation of CRC over healthy controls using Bio-Plex analysis of plasma G-CSF, IFN-G, IL-4, IL-6, IL-8, IL-9, MCP-1, MIP-1B and PDGF-BB in a clinical setting.

### Comparison of Proseek with Bio-plex data

**Figure 4:**
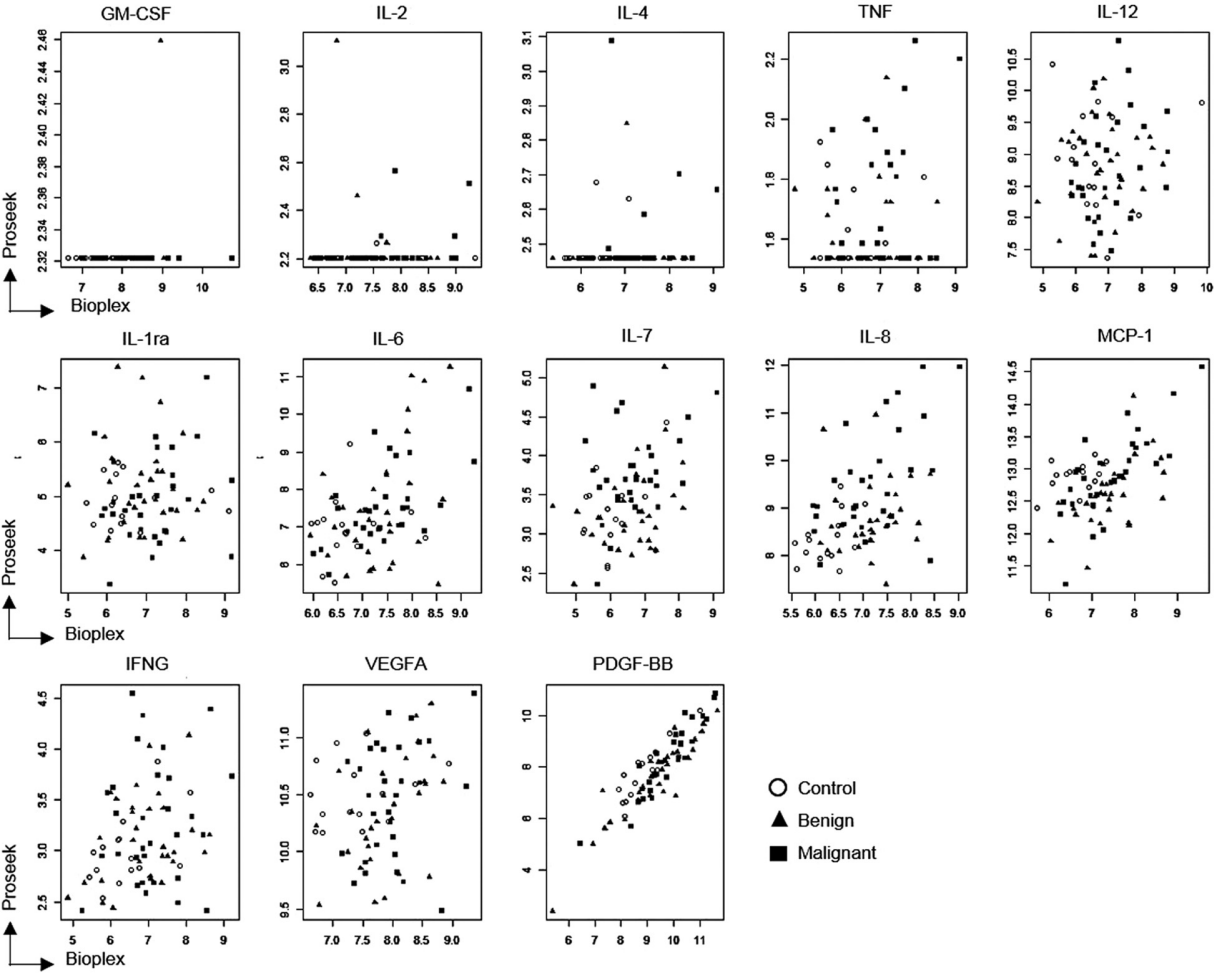
**Spearman correlation scatter plots for 13 individual common biomarkers (PDGF-BB, IL-8, MCP1, IL-6, IL-7, VEGFA, IFNG, IL-2, GM-CSF, IL-4, IL-1ra, IL-12, and TNF) common to the Proseek and Bio-Plex multiplexed immunoassays.**

The 13 common proteins that were available across both the Proseek and Bio-Plex platforms were evaluated by pairing and subsequently analysing the combined data by Spearman’s rank-order correlation (Figure 4). The X-Y comparisons for the Bio-Plex (X) and Proseek (Y) data for these common 13 plasma proteins are provided in Additional file 1: Table S7.

When comparing the two multiplexed immunoassay platforms, there were significant differences between outputs. For a number of proteins (GM-CSF, IL-2, IL-4 and TNF), the correlation was highly skewed as the target biomarker fell below LOD in one or both of the platforms. However, IL-8 and MCP-1 scatter plots suggested there was a positive correlation between data derived from both platforms. The scatter plot data for plasma IL-7, IFN-g, IL-6, VEGF-A levels also suggested moderate correlation. However, the correlation between Proseek and Bio-Plex analyses for PDGF-BB was particularly strong (p and q values = 0) (Additional file 1: Table S7).

In summary, out of the 13 proteins common to both immunoassay platforms, two proteins were identified whose plasma abundance were differentially correlated with Dukes’ CRC clinical stage (IL-8 between samples for Dukes’ D/healthy controls and PDGF-BB between Dukes’ D/A). The remaining eleven proteins did not show major expression difference (*P* > 0.05) across all comparisons made between Dukes’ stage A-D CRC and themselves or healthy controls. For those proteins only available for assay in a single kit, a small number of proteins showed significantly higher expression profiles (8 proteins by Proseek CEA, IL-8, prolactin, amphiregulin, PDGF-BB, IL-6, CXCL11 & CXCL5 and 6 proteins by Bio-Plex: G-CSF, IFN-G, IL-4, IL-8, MCP-1, MIP-1 & PDGF-BB).

Collectively, these differentially expressed plasma proteins epitomise potential Dukes’ stage- and progression-specific CRC biomarkers. The significant differentially expressed proteins identified in this study should now be progressed to a much larger double-blind, multi-centre biomarker trial for further validation of their potential as “combinatorial signatures” of Dukes’ stage-specific CRC.

## Discussion

To the best of our knowledge, this study is one of the first to simultaneously evaluate two independent multiplexed biomarker detection technologies using the same clinical CRC plasma samples. Choi *et al*., undertook an EDTA (Ethylenediaminetetraacetic acid) plasma proteomic study using 2DE/MALDI MS combined with Milliplex MAP Human 26 Plex Cytokine/Chemokine Kit to investigate CRC biomarker signatures [34], but have been heavily criticized for lacking comparison with age- and other criteria-matched healthy controls [35]. In contrast, the current study reports the expression profile of 92 potential oncoproteins biomarkers from patient EDTA plasmas across the four Dukes’ CRC stages combined with an age-, sex-, smoking- and other contraindication-matched healthy group using the recently developed Olink PEA technology. The combined data emanating from the use of this novel multiplexed platform combined with stringent clinical exclusion and inclusion criteria, sample processing and analysis indicates some proteins may be representative of different aspects of progression through Dukes’ staging and potentially reflect real differences in CRC biology *in vivo* during these stages. As such, this study proposes a potential biomarker “signature” of clinical relevance that could be utilised to evaluate Dukes’ CRC stage and progression.

The Proseek® Multiplex Oncology I assay is a high throughput, high sensitivity, specific assay developed for cancer research. It is now realised that the limiting factor in multiplexed immunoassays is antibody cross-reactivity that typically limits the degree of assay multiplexing [36]. Problems with cross-reactivity are virtually eliminated in PEA assays since only matched DNA reporter pairs (i.e., mirroring the presence of two distinct epitopes on the protein) are amplified at the real-time PCR step [12]. Additionally, the small sample volume required (1 μL) to simultaneously quantitatively assay 92 oncoproteins in a multiplexed format is significantly lower than required for alternative platforms (e.g., Luminex-based platforms). This is important as clinical samples are frequently volume limited, particularly when multiple assays (e.g., biomarker “signature” panels) are required or only limited plasma sources (e.g. young children/neonates) are available. As the PEA technology has only recently been commercialized, it was important to validate the platform against an existing benchmark technology (e.g., Bio-Plex multiplex immunoassay) which has FDA approval [37]. The expression profiles of 13 common oncoproteins were compared between both platforms. Nine of those common proteins showed reasonable correlation between platforms, thereby supporting/validating the potential use of the Proseek assay for cancer biomarker research (Figure 4).

Typically immunoassays involve conformational/shape recognition. As such, the knowledge of epitope location or structure is crucial for designing targeted, multiple epitope-based immune assays that can tag multiple sequences for antigen detection. Inadequate mapping can yield false positive or negative results. The Proseek assay uses two proximal epitopes to recognize a single antigen thereby reducing false positive rates which then fall below the LOD (GM-CSF, IL2, IL4 & TNF; Figure 4). However, this may not be a general phenomenon. Proseek technology uses unique sequence-based tagging of every antibody in the assay. By contrast, although the Bio-Plex utilizes a dual antibody system, lack of sequence tagging can create a higher possibility of cross-reactivity and non-specificity [38]. It should be noted that both platforms employ automated software and calibration updates to reduce the need for operator input.

Investigation of individual CRC stage differences in the expression of 92 oncoproteins assessed by the Proseek assay, identified eight oncoproteins that appear to be differentially expressed in plasma as a result of CRC progression (Table 1). CEA, IL-8 and prolactin demonstrated the greatest potential use as diagnostic CRC Dukes’ stage-specific biomarkers (Figure 1). It was interesting to note that except for CEA, none of the other significantly expressed oncoproteins matched with the list of serological CRC indicators identified in a similar study utilizing a 74-plex PEA platform [39]. In that study, Thorsen et al., investigated 74 different protein biomarkers and found carcinoembryonic antigen (CEA), transferrin receptor-1 (TFRC), macrophage migration inhibitory factor (MIF), osteopontin (OPN/SPP1) and cancer antigen 242 (CA242) as CRC discriminators. CEA, TFRC and CA242 were suggested to be early stage CRC indicators. Intriguingly, Choi et al., identified 24 significantly elevated proteins in CRC, of which IL-8, TNF-alpha, and IP-10 (interferon gamma-induced protein) were elevated in the CRC group relative to the adenoma group [34]. Surprisingly, CEA was not a selected as a potential oncoprotein in the Milliplex MAP Human 26 Plex Cytokine/Chemokine Kit used in that study.

CEA is currently employed as a routine marker for CRC prognosis, disease-free survival and therapeutic response [40] and as an independent predictor of patients at higher risk of CRC recurrence and/or metastases during postoperative follow-up [41]. Our study supports aspects of this contention - namely that high plasma CEA expression significantly correlates with the presence of metastatic CRC, though (as previously proposed) it was not found to be an effective plasma biomarker of very early stage disease (Dukes’ A). Another differentially expressed protein (IL-8) is recognized as a pro-inflammatory cytokine and an important chemoattractant factor for leukocytes. IL-8 has been reported to contribute to cancer progression through potential motility-stimulating, mitogenic and angiogenic functions [29]. It has been previously demonstrated that IL-8 is elevated at both the mRNA and plasma protein levels and in CRC tumour tissues compared to adjacent normal colonic mucosa [42-44]. IL-8 is a soluble mediator released by tumor cells that functions within the tumor microenvironment [45]. A number of studies have confirmed the effects of elevated IL-8 on signalling that promotes the angiogenic response and that eventually leads to infiltration of neutrophils to the tumor site [46]. IL-8 expression in tumour tissues significantly correlates with tumour size, depth of infiltration, liver metastasis and tumour stage [24,47]. The present study also confirms that plasma IL-8 concentration significantly discriminates Dukes’ stage D (those with metastatic disease) from either healthy controls or Dukes’ stage A patients.

A number of studies have found that prolactin actively participates in tumorigenesis and that it is overexpressed in several cancer cell lines including those derived from reproductive and non-reproductive tissues [48]. Hence scientists have been interested in developing therapies for controlling tumor growth through suppression of prolactin production [48]. Elevated serum prolactin has been shown to correlate with CRC malignancy [49,50] and is observed in many CRC cell lines and tumour specimens [51,52]. Our data strongly suggests that plasma prolactin significantly correlates with CRC tumour progression through Dukes’ stage A, B and C, being continuously upregulated until distal metastasis occurs. Our data encourage further studies on larger clinical cohorts to evaluate the plasma expression of prolactin during CRC progression as a stage-specific biomarker.

## Conclusions

New, improved and volume-sparing plasma biomarkers/biomarker signature panels are urgently required for cancer screening and surveillance using minimally invasive techniques. Multiplexing represents a powerful platform for the qualitative and quantitative assessment of cancer biomarker signatures, giving the opportunity for sensitive and specific detection of multiple oncoproteins in plasma samples, providing a suite of biomarkers with potential for use as stage-specific indicators of CRC progression.

The emerging PEA technology has received increasing interest considering the large number of target biomarkers that can be measured quantitatively with the advantage of minimum cross-reactivity over benchmark multiplexing platforms. This study has identified eight proteins (CEA, IL-8, prolactin, amphiregulin, PDGF-BB, IL-6, CXCL11 and CXCL5) whose expression trends are of great interest for developing a “biosignatures” of CRC progression that could potentially be translated into a diagnostic/prognostic. Finally, we recognized three prospective novel markers of CRC progression (CEA, IL-8 and prolactin) that hold potential to be utilised in clinical oncology, as they significantly increase with CRC progression and correlate with Dukes’ stage. We recognize the importance of performing further multi-centred large study analysis of marker combinations and in developing new algorithms that confirm improved performance of CEA, perhapsby addition of IL-8 and/or prolactin. As such, we highly recommend the use of these oncoproteins in patient screening as well as for further investigation as potential CRC plasma biomarkers in large multicentric multisample controlled CRC study cohorts.

### Materials & methods

#### Patient plasma samples

Clinically staged CRC (Dukes’ A, B, C, D) and control EDTA-plasma samples were obtained from 75 patients. Patients were Dukes’ staged CRC (15 patients each stage A-D) or apparently healthy disease unaffected controls at Victoria Cancer Biobank (n = 15, called group E subsequently). Samples were stringently age and sex matched with strict inclusion/exclusion criteria applied to minimize variation within the study population. In detail, the study population was a mixture (50:50) of females/males, aged between 50 and 80 for each CRC stage and for the healthy unaffected controls. Samples were collected from CRC patients diagnosed with non-malignant/malignant tumors, before they underwent any treatment and surgery for CRC. The control or unaffected plasma samples were collected from 15 individuals who were aged-matched to the clinical CRC plasma and had no apparent evidence of diseases (i.e., with no evidence of inflammation or metastatic conditions, no previous history of tumor, cancer or major therapy. Clinical details about CRC patients are provided in Additional file 1: Table S8).

### Plasma handling conditions

The samples were collected in 2 EDTA tubes (9 ml each), centrifuged at room temperature (RT) at 1,200 g for 10 mins and plasma fractions transferred to a single 10 ml tube. The combined plasma fractions were centrifuged at RT 1,800 g for 10 mins and aliquoted into 8 × 250 μl tubes that were stored and frozen at -80oC. The entire process was completed within 2 h of plasma collection to meet the VCB protocols required for proteomic experiments.

### Proseek® multiplex oncology I assay

Proseek assays were performed to evaluate the expression of a panel of potential biomarkers within the plasma samples (n = 75) in a 96-well plate. This assay measured 92 potential protein biomarkers (Additional file 1: Table S1) and 4 internal controls generating 9,216 data points per run. The investigation was performed according to manufacturer’s instructions (www.olink.com) with minimal changes. The complete experiment was conducted on a single 96 well plate to minimize variation, with one EDTA plasma sample from each group (number 15) examined in duplicate. Briefly, 1 μl of each sample or negative control was incubated with the conjugated antibodies at 4°C overnight. The next day, the extension mixture was added and the products were extended and pre-amplified using PCR (ABI 2720 Thermal cycler, Life Technologies). The detection reagent and a fraction (2.8 μl) of the extended and pre-amplified product were mixed and loaded into an oil-loaded Fluidigm Gene Expression 96 × 96 Dynamic arrays (Fluidigm Corporation) on one side and the Primer plate with specific primers on the other side of the chip. The chip was primed using Fluidigm IFC controller HX (Fluidigm Corporation) and afterwards loaded into a Fluidigm Biomarker system (Fluidigm Corporation).

Raw data was annotated using Real Time PCR software (Fluidigm Corporation). The Proseek assay generated Cq values that represent the cycle values in the PCR amplification where the signal is above background. This is calculated on a log 2 scale as PCR amplification is increased by 2 fold up during each cycle. To even out variation between and within runs, data was normalized using the extension control and a background value. The data used for further statistical analysis were expressed in Normalized Protein eXpression (NPX) units on log2 scale, where a high value corresponded to high protein concentration. The limit of detection (mean negative control plus 3 × standard deviation) was determined for each protein assay. Data was normalized and analysed using GenEx software (MultiD, Gothenburg, Sweden). All statistical analyses (dynamic principal component analysis and one way ANOVA) were performed on normalized data. For each biomarker, the limit of detection (LOD) was defined as the mean of negative control plus 3 standard deviations of the 38 negative controls.

### Bio-plex Pro™ human cytokine 27-plex immunoassay

Bio-Plex assay (Bio-Rad, CA, USA, Cat No: M500KCAFoY) was utilised according to manufacturer’s instructions and also as reported earlier [23] to measure the concentrations of 27 target proteins (Additional file 1: Table S2) using identical CRC plasma samples as used in the Proseek assay. Samples were prepared using a robotic liquid handling workstation (epMotion 5075, Eppendorf, Germany) and incubated with antibody-coupled beads for 60 min followed by incubation with a detection antibody for 30 min. The conjugates were then incubated with streptavidin for 10 min, washed using the Bio-Plex Pro II wash station (Bio-Rad, CA, USA), resuspended and vortexed prior to fluorescent measurement on a Bio-Plex® 200 system (Bio-Rad, Hercules, CA). Data were acquired with the Bio-Plex Systems 100 (Bio-Rad, CA, USA), analysed and standard curves (Log (x) – Linear(y)) generated using the Bio-Rad Bio-Plex Manager v6.0 software [53].

### Statistical analysis and correlation

Biomarker expression values were analysed using the statistical analytical package RStudio version 0.97.551 across each of stages A-D CRC and E (apparently healthy unaffected controls). In addition, to determine if protein expression could distinguish non-malignant from malignant CRC, individually staged data were pooled into three groups, namely controls (Group E; n = 15), non-malignant CRC (Groups/stage A and B; n = 30) and malignant CRC (Groups/stage C and D; n = 30). This subsequent analysis was performed to determine if significant protein expression differences were found between non-malignant and malignant CRC or when each group was compared to control patients. A two-way ANOVA using type II sums of squares was used for analysis as the control group was now smaller than the combined non-metastatic and metastatic groups implementing an R notation given as: Log2 (Response) ~ Group*Proteins + Sample, where response is the protein expression value, Group is a factor with 3 levels (control, non-metastatic and metastatic), Proteins is a factor with 92 levels for Proseek data set and 27 levels for the Bio-Plex data set and Sample is a factor with 15 levels. Sample represents the plasma samples from CRC patient and unaffected controls that were included in the analysis to allow for any individual differences between patients that might mask the difference in expression levels between CRC stages. P value < 0.05 was considered significant.

To identify which proteins were differentially expressed between collated CRC groups (control, non-malignant and malignant), a Tukey honest significant differences post-hoc test was also performed with respect to the interaction between Protein and Group factors. To discover which proteins exhibited similar expression profiles during CRC progression, self-organizing maps (SOM) were employed to present a discrete representation of the input space of the protein expression values. Raw data was transformed by taking the median expression value for the patient data for each protein within each group. This data was log2 transformed and then each group was normalized by setting each group’s mean value to zero. Expression data was clustered into 6 groups using the SOM and displayed as a plot of the normalized median expressions for each protein with respect to its group values.

To investigate the complementarity of Proseek and Bio-Plex data sets, the expression of 13 proteins that are common to both platforms were paired and analysed by Spearman’s rank-order correlation. This was performed to investigate any potential differences between multiplexed platforms by pairing the expression values and transforming p-values into q-values using the Benjamini and Hochberg procedure for multiple test correction.

## Additional file

Additional file 1: Table S1. List of oncoproteins analyzed by Proseek Assay with Limit of Detection (LOD) in pg/ ml, working range (Lower Limit of Quantification, LLOQ, Upper Limit of Quantification, ULOQ). **Table S2.** List of cytokines analyzed by Bio-plex Assay with Limit of Detection (LOD) in pg/ ml, Lower Limit of Quantification (LLOQ) and Upper Limit of Quantification (ULOQ) (Proteins in bold letters indicate common target proteins between two platforms). **Table S3.** Q- values calculated for stage specific protein expressions analyzed by PEA technology. **Table S4.** Anova Table (Type II tests) or 2-Way Anova factor analysis for Proseek Assay. **Table S5.** p- values calculated for stage specific protein expressions analyzed by Bio-Plex Assay (Stage specific (A-D, n = 15) and healthy group. **Table S6.** Tukey honest significant differences post-hoc test for Bio-plex assay [Group- specific analysis]. **Table S7.** Spearman Correlation between Proseek and Bio-plex assay (with p and q-values). **Table S8.** Clinical Details of CRC patients.
