# Supplementary material for: A novel multiplexed immunoassay identifies CEA, IL-8 and prolactin as prospective markers for Dukes’ stages A-D colorectal cancers
Source: Clin Proteomics. 2015 Apr 8;12(1):10. doi: 10.1186/s12014-015-9081-x (PMC4435647; doi:10.1186/s12014-015-9081-x)
Supplement: Additional file 1: Table S1. — List of oncoproteins analyzed by Proseek Assay with Limit of Detection (LOD) in pg/ ml, working range (Lower Limit of Quantification, LLOQ, Upper Limit of Quantification, ULOQ). Table S2. List of cytokines analyzed by Bio-plex Assay with Limit of Detection (LOD) in pg/ ml, Lower Limit of Quantification (LLOQ) and Upper Limit of Quantification (ULOQ) (Proteins in bold letters indicate common target proteins between two platforms). Table S3. Q- values calculated for stage specific protein expressions analyzed by PEA technology. Table S4. Anova Table (Type II tests) or 2-Way Anova factor analysis for Proseek Assay. Table S5. p- values calculated for stage specific protein expressions analyzed by Bio-Plex Assay (Stage specific (A-D, n = 15) and healthy group. Table S6. Tukey honest significant differences post-hoc test for Bio-plex assay [Group- specific analysis]. Table S7. Spearman Correlation between Proseek and Bio-plex assay (with p and q-values). Table S8. Clinical Details of CRC patients. [file 12014_2015_9081_MOESM1_ESM.docx]

**Supplementary Data**

| **Table S1: List of oncoproteins analyzed by Proseek Assay with Limit of Detection (LOD) in pg/ ml, working range (Lower Limit of Quantification, LLOQ, Upper Limit of Quantification, ULOQ)** | | | | | |
| --- | --- | --- | --- | --- | --- |
| Olink Abbreviations | Gene Name | Target Protein Name | UniProt No | LOD (pg/mL) | Assay Working Range (pg/ml) |
| Adrenomedullin | **ADM** | Adrenomedullin | P35318 | 227.1 | 976.6-250000 |
| Amphiregulin | **AREG** | Amphiregulin | P15514 | 1.58 | 15.3-15625 |
| TIE2 | TIE2 | Angiopoietin-1 receptor | Q02763 | 486.3 | 976.6-250000 |
| BAFF | BAFF | B-cell activating factor | Q9Y275 | 56 | 244.1-250000 |
| Betacellulin | **BTC** | Betacellulin | P35070 | 7.9 | 61.0-3906 |
| CA242 | CA242 | CA242 tumor marker |  | NR | NR-NR |
| CAIX | CAIX | Carbonic Anhydrase IX | Q16790 | 9.9 | 61.0-15625 |
| CEA | CEA | Carcinoembryonic antigen | P06731 | 46.6 | 61.0-62500 |
| Caspase-3 | CASP-3 | Caspase-3 | P42574 | 18.7 | 61.0-62500 |
| Cathepsin D | CTSD | Cathepsin D | P07339 | 6684.7 | 15625.0-1000000 |
| CCL19 | CCL19 | C-C motif chemokine 19 | Q99731 | 5.7 | 15.3-15625 |
| CCL21 | CCL21 | C-C motif chemokine 21 | O00585 | 54.5 | 244.1-3906 |
| CCL24 | CCL24 | C-C motif chemokine 24 | O00175 | 0.44 | 3.8-3906 |
| CD40 ligand | CD40-L | CD40 ligand | P29965 | 0.66 | 3.8-15625 |
| CXCL10 | CXCL10 | C-X-C motif chemokine 10 | P02778 | 4.6 | 15.3-15625 |
| CXCL11 | CXCL11 | C-X-C motif chemokine 11 | O14625 | 14 | 61.0-15625 |
| CXCL13 | CXCL13 | C-X-C motif chemokine 13 | O43927 | 1.16 | 15.3-3906 |
| CXCL5 | CXCL5 | C-X-C motif chemokine 5 | P42830 | 3 | 15.3-15625 |
| CXCL9 | CXCL9 | C-X-C motif chemokine 9 | Q07325 | 4.2 | 61.0-62500 |
| Cystatin B | CPI-B | Cystatin B | P04080 | 207.7 | 976.6-250000 |
| CD69 | CD69 | Benign activation antigen CD69 | Q07108 | 1.15 | 15.3-62500 |
| EGF | EGF | Epidermal growth factor | P01133 | 0.17 | 0.95-3906 |
| EGFR | EGFR | Epidermal growth factor receptor | P00533 | 149.6 | 976.6-250000 |
| HE4 | HE4 | Epididymal secretory protein E4 | Q14508 | 18.8 | 61.0-62500 |
| Epiregulin | EPR | Epiregulin | O14944 | 5.1 | 61.0-15625 |
| Ep-CAM | Ep-CAM | Epithelial cell adhesion molecule | P16422 | 0.44 | 3.8-15625 |
| EPO | EPO | Erythropoietin | P01588 | 81.6 | 244.1-62500 |
| E-selectin | CD62E | E-selectin | P16581 | 1250.5 | 3906.3-250000 |
| Estrogen receptor | ER | Estrogen receptor | P03372 | 375 | 3906.3-250000 |
| EMMPRIN | EMMPRIN | Extracellular matrix metalloproteinase inducer | P35613 | 0.16 | 3.8-15625 |
| FasL | FasL | Fas antigen ligand | P48023 | 2.6 | 15.3-15625 |
| FABP4 | FABP4 | Fatty acid binding protein 4 adipocyte | P15090 | 635.6 | 976.6-250000 |
| Flt3L | Flt3L | Fms-reMalignantd tyrosine kinase 3 ligand | P49771 | 0.18 | 0.95-3906 |
| FR-alpha | FR-alpha | FoMalignant receptor alpha | P15328 | 8.7 | 61.0-62500 |
| Follistatin | FS | Follistatin | P19883 | 31.5 | 244.1-250000 |
| Galectin-3 | Gal-3 | Galectin-3 | P17931 | 3584.5 | 15625.0-1000000 |
| GM-CSF | GM-CSF | Granulocyte-macrophage colonystimulating factor | P04141 | 42 | 244.1-250000 |
| Growth Hormone | GH | Growth Hormone | P01241 | 0.34 | 0.95-15625 |
| GDF-15 | GDF-15 | Growth/differentiation factor 15 | Q99988 | 21.2 | 244.1-62500 |
| HB-EGF | HB-EGF | Heparin-binding EGF-like growth factor | Q99075 | 0.16 | 3.8-3906 |
| HGF | HGF | Hepatocyte growth factor | P14210 | 1.85 | 15.3-15625 |
| HGF receptor | HGF receptor | Hepatocyte growth factor receptor | P08581 | 20.5 | 61.0-62500 |
| IFN-gamma | IFN-gamma | Interferon gamma | P01579 | 14.2 | 61.0-15625 |
| IL-1ra | IL-1ra | Interleukin 1 receptor antagonist protein | P18510 | 105.1 | 244.1-15625 |
| IL-12 | IL-12 | Interleukin 12 | P29460 | 0.77 | 3.8-3906 |
| IL17RB | IL17RB | Interleukin 17 receptor B | Q9NRM | 3.9 | 15.3-62500 |
| IL-2 | IL-2 | Interleukin 2 | P60568 | 55.8 | 244.1-250000 |
| IL2RA | IL2RA | Interleukin 2 receptor subunit alpha | P01589 | 0.19 | 0.95-3906 |
| IL-4 | IL-4 | Interleukin 4 | P05112 | 0.63 | 3.8-15625 |
| IL-6 | IL-6 | Interleukin 6 | P05231 | 0.06 | 0.24-15625 |
| IL6RA | IL6RA | Interleukin 6 receptor subunit alpha | P08887 | 121.2 | 976.6-250000 |
| IL-7 | IL-7 | Interleukin 7 | P13232 | 1.09 | 3.8-15625 |
| IL-8 | IL-8 | Interleukin 8 | P10145 | 0.06 | 0.24-3906 |
| Kallikrein-11 | hK11 | Kallikrein-11 | Q9UBX7 | 145 | 244.1-15625 |
| Kallikrein-6 | KLK6 | Kallikrein-6 | Q92876 | 11 | 61.0-62500 |
| CSF-1 | CSF-1 | Macrophage colony-stimulating factor 1 | P09603 | 0.09 | 0.95-15625 |
| MMP-3 | MMP-3 | Matrix metalloproteinase-3 | P08254 | NR | NR-NR |
| 'MIA | MIA | Melanoma-derived growth regulatory protein | Q16674 | 97.4 | 244.1-62500 |
| MIC-A | MIC-A | MHC class I polypeptide-reMalignantd sequence A | Q29983 | 17.1 | 61.0-3906 |
| Midkine | MK | Midkine | P21741 | 232.9 | 976.6-62500 |
| MCP-1 | MCP-1 | Monocyte chemotactic protein-1 | P13500 | 0.11 | 0.95-3906 |
| MYD88 | MYD88 | Myeloid differentiation primary response protein MyD88 | Q99836 | 69.7 | 244.1-250000 |
| MPO | MPO | Myeloperoxidase | P05164 | 137.8 | 976.6-250000 |
| Osteoprotegerin | OPG | Osteoprotegerin | O00300 | 0.14 | 0.24-15625 |
| CA-125 | CA-125 | Ovarian cancer-reMalignantd tumor marker 125 | Q8WXI7 | NR | NR-NR |
| PlGF | PIGF | Placenta Growth Factor | P49763 | 0.2 | 0.95-15625 |
| PECAM-1 |  | PMalignantlet endothelial cell adhesion molecule | P16284 | 463.4 | 976.6-250000 |
| PDGF subunit B | PDGF subunit B | PMalignantlet-derived growth factor subunit B | P01127 | 92.7 | 244.1-250000 |
| Prolactin | PRL | Prolactin | P01236 | 1925.4 | 3906.3-1000000 |
| Prostasin | PRSS8 | Prostasin | Q16651 | 9.5 | 15.3-15625 |
| PSA | PSA | Prostate-specific antigen | P07288 | 48.6 | 244.1-62500 |
| ErbB2/Her2 | ErbB2/Her2 | Receptor tyrosine-protein kinase ErbB-2 | P04626 | 86.1 | 244.1-62500 |
| ErbB3/Her3 | ErbB3/Her3 | Receptor tyrosine-protein kinase ErbB-3 | P21860 | 16.8 | 61.0-62500 |
| ErbB4/Her4 | ErbB4/Her4 | Receptor tyrosine-protein kinase ErbB-4 | Q15303 | 11.1 | 61.0-62500 |
| REG-4 | REG-4 | Regenerating islet-derived protein 4 | Q9BYZ8 | 259.9 | 3906.3-1000000 |
| Stem cell factor | SCF | Stem cell factor | P21583 | 6.2 | 61.0-62500 |
| TR-AP | TR-AP | Tartrate-resistant acid phosphatase type 5 | P13686 | 58.8 | 244.1-250000 |
| Thrombopoietin | THPO | Thrombopoietin | P40225 | 146.6 | 244.1-62500 |
| Tissue Factor | TF | Tissue Factor | P13726 | 0.81 | 3.8-15625 |
| TGF-alpha | TGF-alpha | Transforming growth factor alpha | P01135 | 0.8 | 3.8-15625 |
| LAP TGF-beta-1 | LAP TGF-beta-1 | Transforming growth factor beta 1 | P01137 | 56.1 | 244.1-250000 |
| TNF | TNF | Tumor necrosis factor alpha | P01375 | 77.4 | 244.1-250000 |
| TNFSF14 | TNFSF14 | Tumor necrosis factor ligand superfamily member 14 | O43557 | 4.5 | 15.3-15625 |
| CD30-L | CD30-L | Tumor necrosis factor ligand superfamily member 8 | P32971 | 9.7 | 61.0-15625 |
| TNF-RI | TNF-RI | Tumor necrosis factor receptor 1 | P19438 | 0.88 | 3.8-15625 |
| TNF-R2 | TNF-R2 | Tumor necrosis factor receptor 2 | P20333 | 247.4 | 976.6-250000 |
| TNFRSF4 | TNFRSF4 | Tumor necrosis factor receptor superfamily member 4 | P43489 | 4.7 | 15.3-15625 |
| FAS | FAS | Tumor necrosis factor receptor superfamily member 6 | P25445 | 12.5 | 244.1-250000 |
| U-PAR | U-PAR | Urokinase plasminogen activator surface receptor | Q03405 | 0.81 | 3.8-15625 |
| VEGF-A | VEGF-A | Vascular endothelial growth factor A | P15692 | 0.32 | 0.95-15625 |
| VEGF-D | VEGF-D | Vascular endothelial growth factor D | O43915 | 22.7 | 61.0-62500 |
| VEGFR-2 | VEGFR-2 | Vascular endothelial growth factor receptor 2 | P35968 | 12.2 | 61.0-62500 |

| **Table S2: List of cytokines analyzed by Bio-plex Assay with Limit of Detection (LOD) in pg/ ml, Lower Limit of Quantification (LLOQ) and Upper Limit of Quantification (ULOQ) (Proteins in bold letters indicate common target proteins between two platforms)** | | | | | |
| --- | --- | --- | --- | --- | --- |
| Cytokine | Gene name | Target Protein Name | UNIPROT ID | LOD | Assay Working range (pg/ml) |
| Eotaxin | CCL11 | C-C motif chemokine 11 | P51671 | 2.5 | 40.9-5,824 |
| FGF basic | FGF2 | Fibroblast growth factor 2 | P09038 | 1.9 | 27.2-7,581 |
| G-CSF | CSF3 | Granulocyte colony-stimulating factor | P09919 | 1.7 | 2.4-11,565 |
| **GM-CSF** | **CSF2** | **Granulocyte-macrophage colony-stimulating factor** | **P04141** | **2.2** | **63.3-6,039** |
| **IFN-gamma** | **IFNG** | **Interferon gamma** | **P01579** | **6.4** | **92.6-52,719** |
| IL-1 beta | IL1B | Interleukin-1 beta | P01584 | 0.6 | 3.2-3,261 |
| **IL-1ra** | **IL1RN** | **Interleukin 1 receptor antagonist protein** | **P18510** | **5.5** | **81.1-70,487** |
| **IL-2** | **IL2** | **Interleukin 2** | **P60568** | **1.6** | **2.1-17,772** |
| **IL-4** | **IL4** | **Interleukin 4** | **P05112** | **0.7** | **2.2-3,467** |
| IL-5 | IL5 | Interleukin-5 | P05113 | 0.6 | 3.1-7,380 |
| **IL-6** | **IL6** | **Interleukin 6** | **P05231** | **2.6** | **2.3-18,880** |
| **IL-7** | **IL7** | **Interleukin 7** | **P13232** | **1.1** | **3.1-6,001** |
| **IL-8** | **IL8** | **Interleukin 8** | **P10145** | **1** | **1.9-26,403** |
| IL-9 | IL9 | Interleukin-9 | P15248 | 2.5 | 2.1-7,989 |
| IL-10 | IL10 | Interleukin-10 | P22301 | 0.3 | 2.2-8,840 |
| **IL12 (p70)** | **IL12** | **Interleukin-12** | **P29460** | **3.5** | **3.3-13,099** |
| IL-13 | IL13 | Interleukin-13 | P35225 | 0.7 | 3.7-3,137 |
| IL-15 | IL15 | Interleukin-15 | P40933 | 2.4 | 2.1-2,799 |
| IL-17 | IL17A | Interleukin-17A | Q16552 | 3.3 | 4.9-12,235 |
| IP-10 | CXCL10 | C-X-C motif chemokine 10 | P02778 | 6.1 | 18.8-26,867 |
| **MCP-1** | **CCL2** | **Monocyte chemotactic protein-1/ C-C motif chemokine 2** | **P13500** | **1.1** | **2.1-1,820** |
| MIP-1 alpha | CCL3 | Macrophage inflammatory protein 1-alpha/ C-C motif chemokine 3 | P10147 | 1.6 | 1.4-836 |
| MIP-1 beta | CCL4 | Macrophage inflammatory protein 1-beta/ C-C motif chemokine 4 | P13236 | 2.4 | 2-1,726 |
| **PDGF-BB** | **PDGFB** | **PMalignantlet-derived growth factor subunit B** | **P01127** | **2.9** | **7-51,933** |
| RANTES | CCL5 | C-C motif chemokine 5 | P13501 | 1.8 | 2.2-8,617 |
| **TNF-alpha** | **TNF** | **Tumor necrosis factor alpha** | **P01375** | **6** | **5.8-95,484** |
| **VEGF** | **VEGFA** | **Vascular endothelial growth factor A** | **P15692** | **3.1** | **5.5-56,237** |

| Table S3: Q- values calculated for stage specific protein expressions analyzed by PEA technology | | | | | |
| --- | --- | --- | --- | --- | --- |
| Candidate Biomarker | Comparison | Difference | Lower CI | Upper CI | Q-value |
| Adrenomedullin | A/E | -0.37343 | -1.66099 | 0.914138 | 1 |
| Adrenomedullin | B/E | 0.042987 | -1.24458 | 1.330551 | 1 |
| Adrenomedullin | C/E | -0.22955 | -1.51712 | 1.058009 | 1 |
| Adrenomedullin | D/E | -0.17962 | -1.46718 | 1.107945 | 1 |
| Adrenomedullin | B/A | 0.416413 | -0.87115 | 1.703976 | 1 |
| Adrenomedullin | C/A | 0.143871 | -1.14369 | 1.431434 | 1 |
| Adrenomedullin | D/A | 0.193806 | -1.09376 | 1.48137 | 1 |
| Adrenomedullin | C/B | -0.27254 | -1.56011 | 1.015022 | 1 |
| Adrenomedullin | D/B | -0.22261 | -1.51017 | 1.064957 | 1 |
| Adrenomedullin | D/C | 0.049936 | -1.23763 | 1.337499 | 1 |
| Amphiregulin | A/E | -0.22732 | -1.51489 | 1.06024 | 1 |
| Amphiregulin | B/E | 0.340724 | -0.94684 | 1.628288 | 1 |
| Amphiregulin | C/E | -0.20858 | -1.49614 | 1.078988 | 1 |
| Amphiregulin | D/E | 1.5605 | 0.272936 | 2.848063 | 0.000236 |
| Amphiregulin | B/A | 0.568048 | -0.71952 | 1.855611 | 1 |
| Amphiregulin | C/A | 0.018748 | -1.26882 | 1.306311 | 1 |
| Amphiregulin | D/A | 1.787823 | 0.50026 | 3.075387 | 8.95E-07 |
| Amphiregulin | C/B | -0.5493 | -1.83686 | 0.738263 | 1 |
| Amphiregulin | D/B | 1.219775 | -0.06779 | 2.507339 | 0.138034 |
| Amphiregulin | D/C | 1.769075 | 0.481512 | 3.056639 | 1.46E-06 |
| BAFF | A/E | -0.42231 | -1.70988 | 0.86525 | 1 |
| BAFF | B/E | -0.22192 | -1.50948 | 1.065645 | 1 |
| BAFF | C/E | -0.50774 | -1.7953 | 0.779828 | 1 |
| BAFF | D/E | 0.044072 | -1.24349 | 1.331635 | 1 |
| BAFF | B/A | 0.200395 | -1.08717 | 1.487958 | 1 |
| BAFF | C/A | -0.08542 | -1.37299 | 1.202141 | 1 |
| BAFF | D/A | 0.466385 | -0.82118 | 1.753948 | 1 |
| BAFF | C/B | -0.28582 | -1.57338 | 1.001746 | 1 |
| BAFF | D/B | 0.26599 | -1.02157 | 1.553553 | 1 |
| BAFF | D/C | 0.551807 | -0.73576 | 1.83937 | 1 |
| Betacellulin | A/E | 0.017737 | -1.26983 | 1.305301 | 1 |
| Betacellulin | B/E | 0.062329 | -1.22523 | 1.349893 | 1 |
| Betacellulin | C/E | -4.22E-15 | -1.28756 | 1.287563 | 1 |
| Betacellulin | D/E | -1.53E-14 | -1.28756 | 1.287563 | 1 |
| Betacellulin | B/A | 0.044592 | -1.24297 | 1.332156 | 1 |
| Betacellulin | C/A | -0.01774 | -1.3053 | 1.269826 | 1 |
| Betacellulin | D/A | -0.01774 | -1.3053 | 1.269826 | 1 |
| Betacellulin | C/B | -0.06233 | -1.34989 | 1.225234 | 1 |
| Betacellulin | D/B | -0.06233 | -1.34989 | 1.225234 | 1 |
| Betacellulin | D/C | -1.11E-14 | -1.28756 | 1.287563 | 1 |
| CA.125 | A/E | -0.32782 | -1.61538 | 0.959745 | 1 |
| CA.125 | B/E | -0.33964 | -1.62721 | 0.947919 | 1 |
| CA.125 | C/E | -0.30166 | -1.58922 | 0.985908 | 1 |
| CA.125 | D/E | -0.00804 | -1.29561 | 1.279521 | 1 |
| CA.125 | B/A | -0.01183 | -1.29939 | 1.275738 | 1 |
| CA.125 | C/A | 0.026163 | -1.2614 | 1.313727 | 1 |
| CA.125 | D/A | 0.319777 | -0.96779 | 1.60734 | 1 |
| CA.125 | C/B | 0.037989 | -1.24957 | 1.325552 | 1 |
| CA.125 | D/B | 0.331602 | -0.95596 | 1.619166 | 1 |
| CA.125 | D/C | 0.293613 | -0.99395 | 1.581177 | 1 |
| CA242 | A/E | 0.180915 | -1.10665 | 1.468478 | 1 |
| CA242 | B/E | 0.048157 | -1.23941 | 1.335721 | 1 |
| CA242 | C/E | 0.148181 | -1.13938 | 1.435744 | 1 |
| CA242 | D/E | 0.557591 | -0.72997 | 1.845154 | 1 |
| CA242 | B/A | -0.13276 | -1.42032 | 1.154806 | 1 |
| CA242 | C/A | -0.03273 | -1.3203 | 1.254829 | 1 |
| CA242 | D/A | 0.376676 | -0.91089 | 1.664239 | 1 |
| CA242 | C/B | 0.100024 | -1.18754 | 1.387587 | 1 |
| CA242 | D/B | 0.509434 | -0.77813 | 1.796997 | 1 |
| CA242 | D/C | 0.40941 | -0.87815 | 1.696973 | 1 |
| CAIX | A/E | 0.065766 | -1.2218 | 1.35333 | 1 |
| CAIX | B/E | 0.182975 | -1.10459 | 1.470539 | 1 |
| CAIX | C/E | -0.01831 | -1.30587 | 1.269255 | 1 |
| CAIX | D/E | 0.884121 | -0.40344 | 2.171685 | 0.998262 |
| CAIX | B/A | 0.117209 | -1.17035 | 1.404772 | 1 |
| CAIX | C/A | -0.08408 | -1.37164 | 1.203488 | 1 |
| CAIX | D/A | 0.818355 | -0.46921 | 2.105919 | 0.999979 |
| CAIX | C/B | -0.20128 | -1.48885 | 1.08628 | 1 |
| CAIX | D/B | 0.701146 | -0.58642 | 1.98871 | 1 |
| CAIX | D/C | 0.90243 | -0.38513 | 2.189994 | 0.995655 |
| Caspase.3 | A/E | -0.30713 | -1.59469 | 0.980435 | 1 |
| Caspase.3 | B/E | 0.333613 | -0.95395 | 1.621176 | 1 |
| Caspase.3 | C/E | 0.114641 | -1.17292 | 1.402205 | 1 |
| Caspase.3 | D/E | 0.670484 | -0.61708 | 1.958048 | 1 |
| Caspase.3 | B/A | 0.640741 | -0.64682 | 1.928305 | 1 |
| Caspase.3 | C/A | 0.42177 | -0.86579 | 1.709333 | 1 |
| Caspase.3 | D/A | 0.977613 | -0.30995 | 2.265176 | 0.935363 |
| Caspase.3 | C/B | -0.21897 | -1.50653 | 1.068592 | 1 |
| Caspase.3 | D/B | 0.336872 | -0.95069 | 1.624435 | 1 |
| Caspase.3 | D/C | 0.555843 | -0.73172 | 1.843406 | 1 |
| Cathepsin.D | A/E | -0.50432 | -1.79188 | 0.783243 | 1 |
| Cathepsin.D | B/E | -0.10476 | -1.39232 | 1.182807 | 1 |
| Cathepsin.D | C/E | -0.364 | -1.65157 | 0.923561 | 1 |
| Cathepsin.D | D/E | -0.24864 | -1.5362 | 1.038927 | 1 |
| Cathepsin.D | B/A | 0.399564 | -0.888 | 1.687128 | 1 |
| Cathepsin.D | C/A | 0.140318 | -1.14725 | 1.427881 | 1 |
| Cathepsin.D | D/A | 0.255684 | -1.03188 | 1.543247 | 1 |
| Cathepsin.D | C/B | -0.25925 | -1.54681 | 1.028317 | 1 |
| Cathepsin.D | D/B | -0.14388 | -1.43144 | 1.143683 | 1 |
| Cathepsin.D | D/C | 0.115366 | -1.1722 | 1.40293 | 1 |
| CCL19 | A/E | -0.67464 | -1.96221 | 0.61292 | 1 |
| CCL19 | B/E | -0.51097 | -1.79853 | 0.776596 | 1 |
| CCL19 | C/E | -0.57792 | -1.86549 | 0.70964 | 1 |
| CCL19 | D/E | -0.27511 | -1.56267 | 1.012454 | 1 |
| CCL19 | B/A | 0.163676 | -1.12389 | 1.451239 | 1 |
| CCL19 | C/A | 0.09672 | -1.19084 | 1.384284 | 1 |
| CCL19 | D/A | 0.399534 | -0.88803 | 1.687097 | 1 |
| CCL19 | C/B | -0.06696 | -1.35452 | 1.220608 | 1 |
| CCL19 | D/B | 0.235858 | -1.05171 | 1.523422 | 1 |
| CCL19 | D/C | 0.302814 | -0.98475 | 1.590377 | 1 |
| CCL21 | A/E | -0.15568 | -1.44324 | 1.131884 | 1 |
| CCL21 | B/E | 0.141274 | -1.14629 | 1.428837 | 1 |
| CCL21 | C/E | -0.18085 | -1.46841 | 1.106717 | 1 |
| CCL21 | D/E | 0.003103 | -1.28446 | 1.290666 | 1 |
| CCL21 | B/A | 0.296953 | -0.99061 | 1.584517 | 1 |
| CCL21 | C/A | -0.02517 | -1.31273 | 1.262396 | 1 |
| CCL21 | D/A | 0.158782 | -1.12878 | 1.446346 | 1 |
| CCL21 | C/B | -0.32212 | -1.60968 | 0.965443 | 1 |
| CCL21 | D/B | -0.13817 | -1.42573 | 1.149392 | 1 |
| CCL21 | D/C | 0.18395 | -1.10361 | 1.471513 | 1 |
| CCL24 | A/E | -0.37707 | -1.66464 | 0.91049 | 1 |
| CCL24 | B/E | -0.52457 | -1.81213 | 0.762996 | 1 |
| CCL24 | C/E | -0.59621 | -1.88377 | 0.691356 | 1 |
| CCL24 | D/E | -0.50964 | -1.7972 | 0.777928 | 1 |
| CCL24 | B/A | -0.14749 | -1.43506 | 1.140069 | 1 |
| CCL24 | C/A | -0.21913 | -1.5067 | 1.068429 | 1 |
| CCL24 | D/A | -0.13256 | -1.42013 | 1.155002 | 1 |
| CCL24 | C/B | -0.07164 | -1.3592 | 1.215923 | 1 |
| CCL24 | D/B | 0.014932 | -1.27263 | 1.302496 | 1 |
| CCL24 | D/C | 0.086572 | -1.20099 | 1.374136 | 1 |
| CD30.L | A/E | 0.208145 | -1.07942 | 1.495708 | 1 |
| CD30.L | B/E | 0.328648 | -0.95892 | 1.616212 | 1 |
| CD30.L | C/E | 0.256961 | -1.0306 | 1.544525 | 1 |
| CD30.L | D/E | 0.315992 | -0.97157 | 1.603555 | 1 |
| CD30.L | B/A | 0.120503 | -1.16706 | 1.408067 | 1 |
| CD30.L | C/A | 0.048816 | -1.23875 | 1.33638 | 1 |
| CD30.L | D/A | 0.107847 | -1.17972 | 1.39541 | 1 |
| CD30.L | C/B | -0.07169 | -1.35925 | 1.215877 | 1 |
| CD30.L | D/B | -0.01266 | -1.30022 | 1.274907 | 1 |
| CD30.L | D/C | 0.05903 | -1.22853 | 1.346594 | 1 |
| CD40.ligand | A/E | -0.51146 | -1.79903 | 0.776101 | 1 |
| CD40.ligand | B/E | -0.22448 | -1.51204 | 1.063083 | 1 |
| CD40.ligand | C/E | -0.44517 | -1.73273 | 0.842397 | 1 |
| CD40.ligand | D/E | 0.327277 | -0.96029 | 1.614841 | 1 |
| CD40.ligand | B/A | 0.286982 | -1.00058 | 1.574545 | 1 |
| CD40.ligand | C/A | 0.066296 | -1.22127 | 1.353859 | 1 |
| CD40.ligand | D/A | 0.838739 | -0.44882 | 2.126303 | 0.999898 |
| CD40.ligand | C/B | -0.22069 | -1.50825 | 1.066877 | 1 |
| CD40.ligand | D/B | 0.551757 | -0.73581 | 1.839321 | 1 |
| CD40.ligand | D/C | 0.772443 | -0.51512 | 2.060007 | 1 |
| CD69 | A/E | -0.08868 | -1.37625 | 1.198881 | 1 |
| CD69 | B/E | 0.550041 | -0.73752 | 1.837604 | 1 |
| CD69 | C/E | 0.281364 | -1.0062 | 1.568927 | 1 |
| CD69 | D/E | 0.574976 | -0.71259 | 1.86254 | 1 |
| CD69 | B/A | 0.638723 | -0.64884 | 1.926286 | 1 |
| CD69 | C/A | 0.370046 | -0.91752 | 1.657609 | 1 |
| CD69 | D/A | 0.663658 | -0.62391 | 1.951222 | 1 |
| CD69 | C/B | -0.26868 | -1.55624 | 1.018886 | 1 |
| CD69 | D/B | 0.024935 | -1.26263 | 1.312499 | 1 |
| CD69 | D/C | 0.293613 | -0.99395 | 1.581176 | 1 |
| CEA | A/E | -0.07688 | -1.36444 | 1.210688 | 1 |
| CEA | B/E | 0.357538 | -0.93003 | 1.645101 | 1 |
| CEA | C/E | 1.317497 | 0.029933 | 2.60506 | 0.030429 |
| CEA | D/E | 2.899803 | 1.612239 | 4.187366 | 1.70E-12 |
| CEA | B/A | 0.434413 | -0.85315 | 1.721976 | 1 |
| CEA | C/A | 1.394372 | 0.106808 | 2.681935 | 0.007576 |
| CEA | D/A | 2.976678 | 1.689114 | 4.264241 | 0 |
| CEA | C/B | 0.959959 | -0.3276 | 2.247522 | 0.960883 |
| CEA | D/B | 2.542265 | 1.254701 | 3.829828 | 4.13E-12 |
| CEA | D/C | 1.582306 | 0.294743 | 2.869869 | 0.000143 |
| CSF.1 | A/E | -0.19372 | -1.48129 | 1.09384 | 1 |
| CSF.1 | B/E | -0.01191 | -1.29947 | 1.275657 | 1 |
| CSF.1 | C/E | -0.35253 | -1.6401 | 0.935029 | 1 |
| CSF.1 | D/E | -0.03133 | -1.3189 | 1.256229 | 1 |
| CSF.1 | B/A | 0.181817 | -1.10575 | 1.469381 | 1 |
| CSF.1 | C/A | -0.15881 | -1.44638 | 1.128752 | 1 |
| CSF.1 | D/A | 0.162389 | -1.12517 | 1.449952 | 1 |
| CSF.1 | C/B | -0.34063 | -1.62819 | 0.946935 | 1 |
| CSF.1 | D/B | -0.01943 | -1.30699 | 1.268135 | 1 |
| CSF.1 | D/C | 0.3212 | -0.96636 | 1.608764 | 1 |
| CXCL10 | A/E | -0.68081 | -1.96837 | 0.606753 | 1 |
| CXCL10 | B/E | -0.47845 | -1.76602 | 0.809112 | 1 |
| CXCL10 | C/E | -0.7874 | -2.07496 | 0.500167 | 0.999999 |
| CXCL10 | D/E | 0.029593 | -1.25797 | 1.317156 | 1 |
| CXCL10 | B/A | 0.202359 | -1.0852 | 1.489922 | 1 |
| CXCL10 | C/A | -0.10659 | -1.39415 | 1.180978 | 1 |
| CXCL10 | D/A | 0.710403 | -0.57716 | 1.997967 | 1 |
| CXCL10 | C/B | -0.30894 | -1.59651 | 0.978619 | 1 |
| CXCL10 | D/B | 0.508045 | -0.77952 | 1.795608 | 1 |
| CXCL10 | D/C | 0.816989 | -0.47057 | 2.104552 | 0.999981 |
| CXCL11 | A/E | -0.48886 | -1.77642 | 0.798705 | 1 |
| CXCL11 | B/E | 0.259621 | -1.02794 | 1.547184 | 1 |
| CXCL11 | C/E | -0.54156 | -1.82912 | 0.746005 | 1 |
| CXCL11 | D/E | 0.814415 | -0.47315 | 2.101979 | 0.999985 |
| CXCL11 | B/A | 0.748479 | -0.53908 | 2.036043 | 1 |
| CXCL11 | C/A | -0.0527 | -1.34026 | 1.234863 | 1 |
| CXCL11 | D/A | 1.303274 | 0.01571 | 2.590837 | 0.038658 |
| CXCL11 | C/B | -0.80118 | -2.08874 | 0.486384 | 0.999995 |
| CXCL11 | D/B | 0.554794 | -0.73277 | 1.842358 | 1 |
| CXCL11 | D/C | 1.355974 | 0.06841 | 2.643537 | 0.015472 |
| CXCL13 | A/E | -0.62111 | -1.90868 | 0.666451 | 1 |
| CXCL13 | B/E | -0.32926 | -1.61682 | 0.958302 | 1 |
| CXCL13 | C/E | -0.06465 | -1.35222 | 1.222911 | 1 |
| CXCL13 | D/E | 0.179172 | -1.10839 | 1.466735 | 1 |
| CXCL13 | B/A | 0.291851 | -0.99571 | 1.579415 | 1 |
| CXCL13 | C/A | 0.556459 | -0.7311 | 1.844023 | 1 |
| CXCL13 | D/A | 0.800284 | -0.48728 | 2.087847 | 0.999996 |
| CXCL13 | C/B | 0.264608 | -1.02296 | 1.552172 | 1 |
| CXCL13 | D/B | 0.508433 | -0.77913 | 1.795996 | 1 |
| CXCL13 | D/C | 0.243825 | -1.04374 | 1.531388 | 1 |
| CXCL5 | A/E | -0.92751 | -2.21508 | 0.360049 | 0.987274 |
| CXCL5 | B/E | -0.13596 | -1.42352 | 1.151606 | 1 |
| CXCL5 | C/E | -0.17444 | -1.462 | 1.113127 | 1 |
| CXCL5 | D/E | 0.370127 | -0.91744 | 1.657691 | 1 |
| CXCL5 | B/A | 0.791557 | -0.49601 | 2.07912 | 0.999998 |
| CXCL5 | C/A | 0.753078 | -0.53449 | 2.040641 | 1 |
| CXCL5 | D/A | 1.297642 | 0.010078 | 2.585205 | 0.04243 |
| CXCL5 | C/B | -0.03848 | -1.32604 | 1.249084 | 1 |
| CXCL5 | D/B | 0.506085 | -0.78148 | 1.793649 | 1 |
| CXCL5 | D/C | 0.544564 | -0.743 | 1.832128 | 1 |
| CXCL9 | A/E | -0.31503 | -1.60259 | 0.972536 | 1 |
| CXCL9 | B/E | 0.305379 | -0.98218 | 1.592943 | 1 |
| CXCL9 | C/E | -0.26451 | -1.55207 | 1.023054 | 1 |
| CXCL9 | D/E | 0.62695 | -0.66061 | 1.914514 | 1 |
| CXCL9 | B/A | 0.620407 | -0.66716 | 1.90797 | 1 |
| CXCL9 | C/A | 0.050518 | -1.23705 | 1.338082 | 1 |
| CXCL9 | D/A | 0.941978 | -0.34559 | 2.229542 | 0.978272 |
| CXCL9 | C/B | -0.56989 | -1.85745 | 0.717675 | 1 |
| CXCL9 | D/B | 0.321571 | -0.96599 | 1.609135 | 1 |
| CXCL9 | D/C | 0.89146 | -0.3961 | 2.179023 | 0.997454 |
| Cystatin.B | A/E | -0.39287 | -1.68043 | 0.894697 | 1 |
| Cystatin.B | B/E | 0.074363 | -1.2132 | 1.361927 | 1 |
| Cystatin.B | C/E | -0.21858 | -1.50615 | 1.06898 | 1 |
| Cystatin.B | D/E | -0.04773 | -1.33529 | 1.239836 | 1 |
| Cystatin.B | B/A | 0.46723 | -0.82033 | 1.754793 | 1 |
| Cystatin.B | C/A | 0.174283 | -1.11328 | 1.461847 | 1 |
| Cystatin.B | D/A | 0.34514 | -0.94242 | 1.632703 | 1 |
| Cystatin.B | C/B | -0.29295 | -1.58051 | 0.994617 | 1 |
| Cystatin.B | D/B | -0.12209 | -1.40965 | 1.165473 | 1 |
| Cystatin.B | D/C | 0.170856 | -1.11671 | 1.45842 | 1 |
| E.selectin | A/E | -0.00044 | -1.288 | 1.287128 | 1 |
| E.selectin | B/E | -0.00074 | -1.28831 | 1.28682 | 1 |
| E.selectin | C/E | -0.29272 | -1.58028 | 0.994846 | 1 |
| E.selectin | D/E | 0.432712 | -0.85485 | 1.720276 | 1 |
| E.selectin | B/A | -0.00031 | -1.28787 | 1.287256 | 1 |
| E.selectin | C/A | -0.29228 | -1.57984 | 0.995282 | 1 |
| E.selectin | D/A | 0.433148 | -0.85442 | 1.720712 | 1 |
| E.selectin | C/B | -0.29197 | -1.57954 | 0.99559 | 1 |
| E.selectin | D/B | 0.433456 | -0.85411 | 1.721019 | 1 |
| E.selectin | D/C | 0.725429 | -0.56213 | 2.012993 | 1 |
| EGF | A/E | -0.54822 | -1.83578 | 0.739342 | 1 |
| EGF | B/E | 0.081633 | -1.20593 | 1.369197 | 1 |
| EGF | C/E | -0.15077 | -1.43834 | 1.13679 | 1 |
| EGF | D/E | 0.480911 | -0.80665 | 1.768474 | 1 |
| EGF | B/A | 0.629854 | -0.65771 | 1.917418 | 1 |
| EGF | C/A | 0.397448 | -0.89012 | 1.685011 | 1 |
| EGF | D/A | 1.029132 | -0.25843 | 2.316695 | 0.803702 |
| EGF | C/B | -0.23241 | -1.51997 | 1.055157 | 1 |
| EGF | D/B | 0.399277 | -0.88829 | 1.686841 | 1 |
| EGF | D/C | 0.631684 | -0.65588 | 1.919247 | 1 |
| EGFR | A/E | -0.3328 | -1.62037 | 0.954759 | 1 |
| EGFR | B/E | -0.40615 | -1.69371 | 0.881415 | 1 |
| EGFR | C/E | -0.41663 | -1.70419 | 0.870933 | 1 |
| EGFR | D/E | -0.24693 | -1.53449 | 1.040633 | 1 |
| EGFR | B/A | -0.07334 | -1.36091 | 1.21422 | 1 |
| EGFR | C/A | -0.08383 | -1.37139 | 1.203738 | 1 |
| EGFR | D/A | 0.085875 | -1.20169 | 1.373438 | 1 |
| EGFR | C/B | -0.01048 | -1.29805 | 1.277081 | 1 |
| EGFR | D/B | 0.159218 | -1.12835 | 1.446781 | 1 |
| EGFR | D/C | 0.1697 | -1.11786 | 1.457264 | 1 |
| EMMPRIN | A/E | 0.320567 | -0.967 | 1.60813 | 1 |
| EMMPRIN | B/E | -0.15027 | -1.43783 | 1.137292 | 1 |
| EMMPRIN | C/E | 0.34046 | -0.9471 | 1.628023 | 1 |
| EMMPRIN | D/E | 0.333331 | -0.95423 | 1.620895 | 1 |
| EMMPRIN | B/A | -0.47084 | -1.7584 | 0.816725 | 1 |
| EMMPRIN | C/A | 0.019893 | -1.26767 | 1.307456 | 1 |
| EMMPRIN | D/A | 0.012764 | -1.2748 | 1.300328 | 1 |
| EMMPRIN | C/B | 0.490731 | -0.79683 | 1.778294 | 1 |
| EMMPRIN | D/B | 0.483602 | -0.80396 | 1.771166 | 1 |
| EMMPRIN | D/C | -0.00713 | -1.29469 | 1.280435 | 1 |
| Ep.CAM | A/E | -0.48613 | -1.7737 | 0.801431 | 1 |
| Ep.CAM | B/E | -0.43517 | -1.72274 | 0.852389 | 1 |
| Ep.CAM | C/E | -0.35947 | -1.64704 | 0.928091 | 1 |
| Ep.CAM | D/E | 0.421201 | -0.86636 | 1.708765 | 1 |
| Ep.CAM | B/A | 0.050958 | -1.23661 | 1.338521 | 1 |
| Ep.CAM | C/A | 0.12666 | -1.1609 | 1.414224 | 1 |
| Ep.CAM | D/A | 0.907334 | -0.38023 | 2.194897 | 0.994555 |
| Ep.CAM | C/B | 0.075703 | -1.21186 | 1.363266 | 1 |
| Ep.CAM | D/B | 0.856376 | -0.43119 | 2.143939 | 0.999659 |
| Ep.CAM | D/C | 0.780673 | -0.50689 | 2.068237 | 0.999999 |
| Epiregulin | A/E | 3.33E-14 | -1.28756 | 1.287563 | 1 |
| Epiregulin | B/E | 1.27E-14 | -1.28756 | 1.287563 | 1 |
| Epiregulin | C/E | 0.010219 | -1.27734 | 1.297782 | 1 |
| Epiregulin | D/E | 0.027725 | -1.25984 | 1.315289 | 1 |
| Epiregulin | B/A | -2.07E-14 | -1.28756 | 1.287563 | 1 |
| Epiregulin | C/A | 0.010219 | -1.27734 | 1.297782 | 1 |
| Epiregulin | D/A | 0.027725 | -1.25984 | 1.315289 | 1 |
| Epiregulin | C/B | 0.010219 | -1.27734 | 1.297782 | 1 |
| Epiregulin | D/B | 0.027725 | -1.25984 | 1.315289 | 1 |
| Epiregulin | D/C | 0.017507 | -1.27006 | 1.30507 | 1 |
| EPO | A/E | -0.14463 | -1.43219 | 1.142938 | 1 |
| EPO | B/E | 0.188557 | -1.09901 | 1.47612 | 1 |
| EPO | C/E | -0.06631 | -1.35387 | 1.221252 | 1 |
| EPO | D/E | -0.20288 | -1.49044 | 1.084683 | 1 |
| EPO | B/A | 0.333182 | -0.95438 | 1.620746 | 1 |
| EPO | C/A | 0.078314 | -1.20925 | 1.365878 | 1 |
| EPO | D/A | -0.05826 | -1.34582 | 1.229308 | 1 |
| EPO | C/B | -0.25487 | -1.54243 | 1.032696 | 1 |
| EPO | D/B | -0.39144 | -1.679 | 0.896126 | 1 |
| EPO | D/C | -0.13657 | -1.42413 | 1.150994 | 1 |
| ErbB2.Her2 | A/E | -0.15048 | -1.43805 | 1.137081 | 1 |
| ErbB2.Her2 | B/E | -0.23611 | -1.52368 | 1.05145 | 1 |
| ErbB2.Her2 | C/E | -0.38065 | -1.66822 | 0.906911 | 1 |
| ErbB2.Her2 | D/E | -0.09415 | -1.38171 | 1.193415 | 1 |
| ErbB2.Her2 | B/A | -0.08563 | -1.37319 | 1.201933 | 1 |
| ErbB2.Her2 | C/A | -0.23017 | -1.51773 | 1.057393 | 1 |
| ErbB2.Her2 | D/A | 0.056334 | -1.23123 | 1.343897 | 1 |
| ErbB2.Her2 | C/B | -0.14454 | -1.4321 | 1.143024 | 1 |
| ErbB2.Her2 | D/B | 0.141965 | -1.1456 | 1.429528 | 1 |
| ErbB2.Her2 | D/C | 0.286504 | -1.00106 | 1.574067 | 1 |
| ErbB3.Her3 | A/E | -0.20242 | -1.48998 | 1.085144 | 1 |
| ErbB3.Her3 | B/E | -0.22752 | -1.51509 | 1.060041 | 1 |
| ErbB3.Her3 | C/E | -0.21303 | -1.5006 | 1.074529 | 1 |
| ErbB3.Her3 | D/E | -0.13316 | -1.42072 | 1.154407 | 1 |
| ErbB3.Her3 | B/A | -0.0251 | -1.31267 | 1.262461 | 1 |
| ErbB3.Her3 | C/A | -0.01061 | -1.29818 | 1.276949 | 1 |
| ErbB3.Her3 | D/A | 0.069264 | -1.2183 | 1.356827 | 1 |
| ErbB3.Her3 | C/B | 0.014488 | -1.27308 | 1.302051 | 1 |
| ErbB3.Her3 | D/B | 0.094366 | -1.1932 | 1.38193 | 1 |
| ErbB3.Her3 | D/C | 0.079878 | -1.20768 | 1.367442 | 1 |
| ErbB4.Her4 | A/E | -0.39023 | -1.67779 | 0.897332 | 1 |
| ErbB4.Her4 | B/E | -0.40493 | -1.6925 | 0.882632 | 1 |
| ErbB4.Her4 | C/E | -0.4994 | -1.78696 | 0.788164 | 1 |
| ErbB4.Her4 | D/E | -0.38354 | -1.6711 | 0.904022 | 1 |
| ErbB4.Her4 | B/A | -0.0147 | -1.30226 | 1.272863 | 1 |
| ErbB4.Her4 | C/A | -0.10917 | -1.39673 | 1.178395 | 1 |
| ErbB4.Her4 | D/A | 0.00669 | -1.28087 | 1.294253 | 1 |
| ErbB4.Her4 | C/B | -0.09447 | -1.38203 | 1.193096 | 1 |
| ErbB4.Her4 | D/B | 0.02139 | -1.26617 | 1.308954 | 1 |
| ErbB4.Her4 | D/C | 0.115858 | -1.17171 | 1.403421 | 1 |
| Estrogen.receptor | A/E | 2.31E-14 | -1.28756 | 1.287563 | 1 |
| Estrogen.receptor | B/E | 2.22E-15 | -1.28756 | 1.287563 | 1 |
| Estrogen.receptor | C/E | 0.003838 | -1.28373 | 1.291401 | 1 |
| Estrogen.receptor | D/E | 0.011243 | -1.27632 | 1.298807 | 1 |
| Estrogen.receptor | B/A | -2.09E-14 | -1.28756 | 1.287563 | 1 |
| Estrogen.receptor | C/A | 0.003838 | -1.28373 | 1.291401 | 1 |
| Estrogen.receptor | D/A | 0.011243 | -1.27632 | 1.298807 | 1 |
| Estrogen.receptor | C/B | 0.003838 | -1.28373 | 1.291401 | 1 |
| Estrogen.receptor | D/B | 0.011243 | -1.27632 | 1.298807 | 1 |
| Estrogen.receptor | D/C | 0.007406 | -1.28016 | 1.294969 | 1 |
| FABP4 | A/E | -0.02496 | -1.31252 | 1.262608 | 1 |
| FABP4 | B/E | 0.213661 | -1.0739 | 1.501225 | 1 |
| FABP4 | C/E | 0.220405 | -1.06716 | 1.507969 | 1 |
| FABP4 | D/E | -0.22565 | -1.51321 | 1.061914 | 1 |
| FABP4 | B/A | 0.238617 | -1.04895 | 1.52618 | 1 |
| FABP4 | C/A | 0.245361 | -1.0422 | 1.532924 | 1 |
| FABP4 | D/A | -0.20069 | -1.48826 | 1.086869 | 1 |
| FABP4 | C/B | 0.006744 | -1.28082 | 1.294307 | 1 |
| FABP4 | D/B | -0.43931 | -1.72687 | 0.848253 | 1 |
| FABP4 | D/C | -0.44605 | -1.73362 | 0.841509 | 1 |
| FAS | A/E | -0.36378 | -1.65134 | 0.923786 | 1 |
| FAS | B/E | -0.20395 | -1.49151 | 1.083614 | 1 |
| FAS | C/E | -0.34914 | -1.6367 | 0.938426 | 1 |
| FAS | D/E | -0.18012 | -1.46768 | 1.107444 | 1 |
| FAS | B/A | 0.159829 | -1.12773 | 1.447392 | 1 |
| FAS | C/A | 0.01464 | -1.27292 | 1.302204 | 1 |
| FAS | D/A | 0.183658 | -1.10391 | 1.471221 | 1 |
| FAS | C/B | -0.14519 | -1.43275 | 1.142375 | 1 |
| FAS | D/B | 0.023829 | -1.26373 | 1.311393 | 1 |
| FAS | D/C | 0.169018 | -1.11855 | 1.456581 | 1 |
| FasL | A/E | -0.03079 | -1.31835 | 1.256778 | 1 |
| FasL | B/E | -0.04012 | -1.32768 | 1.247448 | 1 |
| FasL | C/E | 0.049413 | -1.23815 | 1.336976 | 1 |
| FasL | D/E | 0.001596 | -1.28597 | 1.28916 | 1 |
| FasL | B/A | -0.00933 | -1.29689 | 1.278233 | 1 |
| FasL | C/A | 0.080198 | -1.20737 | 1.367761 | 1 |
| FasL | D/A | 0.032381 | -1.25518 | 1.319945 | 1 |
| FasL | C/B | 0.089528 | -1.19804 | 1.377092 | 1 |
| FasL | D/B | 0.041712 | -1.24585 | 1.329275 | 1 |
| FasL | D/C | -0.04782 | -1.33538 | 1.239747 | 1 |
| Flt3L | A/E | -0.49858 | -1.78614 | 0.788983 | 1 |
| Flt3L | B/E | -0.2944 | -1.58197 | 0.993161 | 1 |
| Flt3L | C/E | -0.59173 | -1.8793 | 0.695832 | 1 |
| Flt3L | D/E | -0.30745 | -1.59502 | 0.980109 | 1 |
| Flt3L | B/A | 0.204178 | -1.08339 | 1.491742 | 1 |
| Flt3L | C/A | -0.09315 | -1.38071 | 1.194412 | 1 |
| Flt3L | D/A | 0.191126 | -1.09644 | 1.478689 | 1 |
| Flt3L | C/B | -0.29733 | -1.58489 | 0.990234 | 1 |
| Flt3L | D/B | -0.01305 | -1.30062 | 1.274511 | 1 |
| Flt3L | D/C | 0.284277 | -1.00329 | 1.571841 | 1 |
| Follistatin | A/E | -0.20524 | -1.49281 | 1.082319 | 1 |
| Follistatin | B/E | 0.316564 | -0.971 | 1.604127 | 1 |
| Follistatin | C/E | 0.137673 | -1.14989 | 1.425237 | 1 |
| Follistatin | D/E | 0.056123 | -1.23144 | 1.343686 | 1 |
| Follistatin | B/A | 0.521808 | -0.76576 | 1.809372 | 1 |
| Follistatin | C/A | 0.342918 | -0.94465 | 1.630482 | 1 |
| Follistatin | D/A | 0.261367 | -1.0262 | 1.548931 | 1 |
| Follistatin | C/B | -0.17889 | -1.46645 | 1.108673 | 1 |
| Follistatin | D/B | -0.26044 | -1.548 | 1.027123 | 1 |
| Follistatin | D/C | -0.08155 | -1.36911 | 1.206013 | 1 |
| FR.alpha | A/E | -0.3325 | -1.62006 | 0.955062 | 1 |
| FR.alpha | B/E | -0.12797 | -1.41553 | 1.159598 | 1 |
| FR.alpha | C/E | -0.35417 | -1.64173 | 0.933396 | 1 |
| FR.alpha | D/E | -0.32852 | -1.61609 | 0.95904 | 1 |
| FR.alpha | B/A | 0.204536 | -1.08303 | 1.492099 | 1 |
| FR.alpha | C/A | -0.02167 | -1.30923 | 1.265897 | 1 |
| FR.alpha | D/A | 0.003978 | -1.28359 | 1.291541 | 1 |
| FR.alpha | C/B | -0.2262 | -1.51377 | 1.061361 | 1 |
| FR.alpha | D/B | -0.20056 | -1.48812 | 1.087006 | 1 |
| FR.alpha | D/C | 0.025644 | -1.26192 | 1.313208 | 1 |
| Galectin.3 | A/E | -0.39005 | -1.67761 | 0.897516 | 1 |
| Galectin.3 | B/E | -0.23158 | -1.51915 | 1.05598 | 1 |
| Galectin.3 | C/E | -0.20829 | -1.49585 | 1.079276 | 1 |
| Galectin.3 | D/E | 0.23005 | -1.05751 | 1.517614 | 1 |
| Galectin.3 | B/A | 0.158464 | -1.1291 | 1.446028 | 1 |
| Galectin.3 | C/A | 0.18176 | -1.1058 | 1.469323 | 1 |
| Galectin.3 | D/A | 0.620098 | -0.66747 | 1.907661 | 1 |
| Galectin.3 | C/B | 0.023295 | -1.26427 | 1.310859 | 1 |
| Galectin.3 | D/B | 0.461633 | -0.82593 | 1.749197 | 1 |
| Galectin.3 | D/C | 0.438338 | -0.84923 | 1.725902 | 1 |
| GDF.15 | A/E | -0.36566 | -1.65322 | 0.921907 | 1 |
| GDF.15 | B/E | 0.69645 | -0.59111 | 1.984014 | 1 |
| GDF.15 | C/E | 0.075028 | -1.21254 | 1.362591 | 1 |
| GDF.15 | D/E | 0.649793 | -0.63777 | 1.937357 | 1 |
| GDF.15 | B/A | 1.062107 | -0.22546 | 2.34967 | 0.679682 |
| GDF.15 | C/A | 0.440684 | -0.84688 | 1.728248 | 1 |
| GDF.15 | D/A | 1.01545 | -0.27211 | 2.303013 | 0.847039 |
| GDF.15 | C/B | -0.62142 | -1.90899 | 0.666141 | 1 |
| GDF.15 | D/B | -0.04666 | -1.33422 | 1.240906 | 1 |
| GDF.15 | D/C | 0.574766 | -0.7128 | 1.862329 | 1 |
| GM.CSF | A/E | 0.008594 | -1.27897 | 1.296157 | 1 |
| GM.CSF | B/E | -3.55E-15 | -1.28756 | 1.287563 | 1 |
| GM.CSF | C/E | -1.78E-15 | -1.28756 | 1.287563 | 1 |
| GM.CSF | D/E | -1.24E-14 | -1.28756 | 1.287563 | 1 |
| GM.CSF | B/A | -0.00859 | -1.29616 | 1.278969 | 1 |
| GM.CSF | C/A | -0.00859 | -1.29616 | 1.278969 | 1 |
| GM.CSF | D/A | -0.00859 | -1.29616 | 1.278969 | 1 |
| GM.CSF | C/B | 1.78E-15 | -1.28756 | 1.287563 | 1 |
| GM.CSF | D/B | -8.88E-15 | -1.28756 | 1.287563 | 1 |
| GM.CSF | D/C | -1.07E-14 | -1.28756 | 1.287563 | 1 |
| Growth.Hormone | A/E | 0.729487 | -0.55808 | 2.017051 | 1 |
| Growth.Hormone | B/E | 0.576355 | -0.71121 | 1.863919 | 1 |
| Growth.Hormone | C/E | 0.79832 | -0.48924 | 2.085883 | 0.999996 |
| Growth.Hormone | D/E | 0.362469 | -0.92509 | 1.650033 | 1 |
| Growth.Hormone | B/A | -0.15313 | -1.4407 | 1.134431 | 1 |
| Growth.Hormone | C/A | 0.068832 | -1.21873 | 1.356396 | 1 |
| Growth.Hormone | D/A | -0.36702 | -1.65458 | 0.920545 | 1 |
| Growth.Hormone | C/B | 0.221965 | -1.0656 | 1.509528 | 1 |
| Growth.Hormone | D/B | -0.21389 | -1.50145 | 1.073677 | 1 |
| Growth.Hormone | D/C | -0.43585 | -1.72341 | 0.851713 | 1 |
| HB.EGF | A/E | -0.20058 | -1.48814 | 1.086988 | 1 |
| HB.EGF | B/E | -0.00285 | -1.29041 | 1.284715 | 1 |
| HB.EGF | C/E | -0.2012 | -1.48876 | 1.086365 | 1 |
| HB.EGF | D/E | 0.358139 | -0.92942 | 1.645702 | 1 |
| HB.EGF | B/A | 0.197728 | -1.08984 | 1.485291 | 1 |
| HB.EGF | C/A | -0.00062 | -1.28819 | 1.286941 | 1 |
| HB.EGF | D/A | 0.558715 | -0.72885 | 1.846278 | 1 |
| HB.EGF | C/B | -0.19835 | -1.48591 | 1.089213 | 1 |
| HB.EGF | D/B | 0.360987 | -0.92658 | 1.64855 | 1 |
| HB.EGF | D/C | 0.559337 | -0.72823 | 1.8469 | 1 |
| HE4 | A/E | -0.20184 | -1.4894 | 1.085728 | 1 |
| HE4 | B/E | -0.0913 | -1.37886 | 1.196264 | 1 |
| HE4 | C/E | -0.16483 | -1.45239 | 1.122736 | 1 |
| HE4 | D/E | -0.10764 | -1.39521 | 1.179919 | 1 |
| HE4 | B/A | 0.110536 | -1.17703 | 1.398099 | 1 |
| HE4 | C/A | 0.037009 | -1.25055 | 1.324572 | 1 |
| HE4 | D/A | 0.094192 | -1.19337 | 1.381755 | 1 |
| HE4 | C/B | -0.07353 | -1.36109 | 1.214036 | 1 |
| HE4 | D/B | -0.01634 | -1.30391 | 1.271219 | 1 |
| HE4 | D/C | 0.057183 | -1.23038 | 1.344747 | 1 |
| HGF | A/E | 0.018823 | -1.26874 | 1.306387 | 1 |
| HGF | B/E | 0.532771 | -0.75479 | 1.820335 | 1 |
| HGF | C/E | -0.34026 | -1.62783 | 0.947301 | 1 |
| HGF | D/E | -0.06215 | -1.34972 | 1.22541 | 1 |
| HGF | B/A | 0.513948 | -0.77362 | 1.801511 | 1 |
| HGF | C/A | -0.35909 | -1.64665 | 0.928477 | 1 |
| HGF | D/A | -0.08098 | -1.36854 | 1.206587 | 1 |
| HGF | C/B | -0.87303 | -2.1606 | 0.41453 | 0.99906 |
| HGF | D/B | -0.59492 | -1.88249 | 0.692639 | 1 |
| HGF | D/C | 0.27811 | -1.00945 | 1.565673 | 1 |
| HGF.receptor | A/E | -0.02215 | -1.30972 | 1.26541 | 1 |
| HGF.receptor | B/E | -0.1043 | -1.39186 | 1.183263 | 1 |
| HGF.receptor | C/E | -0.23358 | -1.52115 | 1.05398 | 1 |
| HGF.receptor | D/E | -0.11026 | -1.39783 | 1.177301 | 1 |
| HGF.receptor | B/A | -0.08215 | -1.36971 | 1.205417 | 1 |
| HGF.receptor | C/A | -0.21143 | -1.49899 | 1.076133 | 1 |
| HGF.receptor | D/A | -0.08811 | -1.37567 | 1.199454 | 1 |
| HGF.receptor | C/B | -0.12928 | -1.41685 | 1.15828 | 1 |
| HGF.receptor | D/B | -0.00596 | -1.29353 | 1.281601 | 1 |
| HGF.receptor | D/C | 0.123321 | -1.16424 | 1.410885 | 1 |
| IFN.gamma | A/E | 0.06658 | -1.22098 | 1.354144 | 1 |
| IFN.gamma | B/E | 0.091151 | -1.19641 | 1.378715 | 1 |
| IFN.gamma | C/E | 0.25105 | -1.03651 | 1.538613 | 1 |
| IFN.gamma | D/E | 0.063843 | -1.22372 | 1.351406 | 1 |
| IFN.gamma | B/A | 0.024571 | -1.26299 | 1.312134 | 1 |
| IFN.gamma | C/A | 0.184469 | -1.10309 | 1.472032 | 1 |
| IFN.gamma | D/A | -0.00274 | -1.2903 | 1.284826 | 1 |
| IFN.gamma | C/B | 0.159898 | -1.12767 | 1.447462 | 1 |
| IFN.gamma | D/B | -0.02731 | -1.31487 | 1.260255 | 1 |
| IFN.gamma | D/C | -0.18721 | -1.47477 | 1.100357 | 1 |
| IL.12 | A/E | -0.41984 | -1.7074 | 0.867725 | 1 |
| IL.12 | B/E | 0.306472 | -0.98109 | 1.594036 | 1 |
| IL.12 | C/E | -0.13656 | -1.42412 | 1.151007 | 1 |
| IL.12 | D/E | -0.1022 | -1.38976 | 1.185365 | 1 |
| IL.12 | B/A | 0.726311 | -0.56125 | 2.013875 | 1 |
| IL.12 | C/A | 0.283283 | -1.00428 | 1.570846 | 1 |
| IL.12 | D/A | 0.31764 | -0.96992 | 1.605204 | 1 |
| IL.12 | C/B | -0.44303 | -1.73059 | 0.844535 | 1 |
| IL.12 | D/B | -0.40867 | -1.69623 | 0.878892 | 1 |
| IL.12 | D/C | 0.034357 | -1.25321 | 1.321921 | 1 |
| IL.1ra | A/E | 0.231602 | -1.05596 | 1.519165 | 1 |
| IL.1ra | B/E | 0.401115 | -0.88645 | 1.688678 | 1 |
| IL.1ra | C/E | -0.29876 | -1.58632 | 0.988808 | 1 |
| IL.1ra | D/E | 0.279447 | -1.00812 | 1.56701 | 1 |
| IL.1ra | B/A | 0.169513 | -1.11805 | 1.457077 | 1 |
| IL.1ra | C/A | -0.53036 | -1.81792 | 0.757207 | 1 |
| IL.1ra | D/A | 0.047845 | -1.23972 | 1.335409 | 1 |
| IL.1ra | C/B | -0.69987 | -1.98743 | 0.587694 | 1 |
| IL.1ra | D/B | -0.12167 | -1.40923 | 1.165895 | 1 |
| IL.1ra | D/C | 0.578202 | -0.70936 | 1.865765 | 1 |
| IL.2 | A/E | -0.00384 | -1.2914 | 1.283726 | 1 |
| IL.2 | B/E | 0.072531 | -1.21503 | 1.360095 | 1 |
| IL.2 | C/E | 0.037938 | -1.24963 | 1.325501 | 1 |
| IL.2 | D/E | 0.007556 | -1.28001 | 1.295119 | 1 |
| IL.2 | B/A | 0.076369 | -1.21119 | 1.363932 | 1 |
| IL.2 | C/A | 0.041776 | -1.24579 | 1.329339 | 1 |
| IL.2 | D/A | 0.011393 | -1.27617 | 1.298957 | 1 |
| IL.2 | C/B | -0.03459 | -1.32216 | 1.25297 | 1 |
| IL.2 | D/B | -0.06498 | -1.35254 | 1.222588 | 1 |
| IL.2 | D/C | -0.03038 | -1.31795 | 1.257181 | 1 |
| IL.4 | A/E | -0.0338 | -1.32137 | 1.25376 | 1 |
| IL.4 | B/E | -0.00952 | -1.29708 | 1.278045 | 1 |
| IL.4 | C/E | 0.007073 | -1.28049 | 1.294637 | 1 |
| IL.4 | D/E | 0.00135 | -1.28621 | 1.288914 | 1 |
| IL.4 | B/A | 0.024285 | -1.26328 | 1.311849 | 1 |
| IL.4 | C/A | 0.040877 | -1.24669 | 1.32844 | 1 |
| IL.4 | D/A | 0.035154 | -1.25241 | 1.322717 | 1 |
| IL.4 | C/B | 0.016591 | -1.27097 | 1.304155 | 1 |
| IL.4 | D/B | 0.010868 | -1.2767 | 1.298432 | 1 |
| IL.4 | D/C | -0.00572 | -1.29329 | 1.28184 | 1 |
| IL.6 | A/E | -0.08436 | -1.37193 | 1.203201 | 1 |
| IL.6 | B/E | 1.368284 | 0.080721 | 2.655848 | 0.012356 |
| IL.6 | C/E | 0.270008 | -1.01756 | 1.557571 | 1 |
| IL.6 | D/E | 0.75137 | -0.53619 | 2.038933 | 1 |
| IL.6 | B/A | 1.452647 | 0.165083 | 2.74021 | 0.002396 |
| IL.6 | C/A | 0.35437 | -0.93319 | 1.641933 | 1 |
| IL.6 | D/A | 0.835732 | -0.45183 | 2.123295 | 0.999918 |
| IL.6 | C/B | -1.09828 | -2.38584 | 0.189287 | 0.527659 |
| IL.6 | D/B | -0.61691 | -1.90448 | 0.670649 | 1 |
| IL.6 | D/C | 0.481362 | -0.8062 | 1.768925 | 1 |
| IL.7 | A/E | -0.18069 | -1.46826 | 1.10687 | 1 |
| IL.7 | B/E | 0.150554 | -1.13701 | 1.438117 | 1 |
| IL.7 | C/E | 0.346557 | -0.94101 | 1.634121 | 1 |
| IL.7 | D/E | 0.491123 | -0.79644 | 1.778686 | 1 |
| IL.7 | B/A | 0.331247 | -0.95632 | 1.618811 | 1 |
| IL.7 | C/A | 0.527251 | -0.76031 | 1.814814 | 1 |
| IL.7 | D/A | 0.671816 | -0.61575 | 1.95938 | 1 |
| IL.7 | C/B | 0.196004 | -1.09156 | 1.483567 | 1 |
| IL.7 | D/B | 0.340569 | -0.94699 | 1.628132 | 1 |
| IL.7 | D/C | 0.144565 | -1.143 | 1.432129 | 1 |
| IL.8 | A/E | 0.126378 | -1.16119 | 1.413941 | 1 |
| IL.8 | B/E | 0.693238 | -0.59432 | 1.980802 | 1 |
| IL.8 | C/E | 0.668738 | -0.61883 | 1.956301 | 1 |
| IL.8 | D/E | 1.775656 | 0.488093 | 3.06322 | 1.23E-06 |
| IL.8 | B/A | 0.56686 | -0.7207 | 1.854424 | 1 |
| IL.8 | C/A | 0.54236 | -0.7452 | 1.829923 | 1 |
| IL.8 | D/A | 1.649278 | 0.361715 | 2.936842 | 2.96E-05 |
| IL.8 | C/B | -0.0245 | -1.31206 | 1.263063 | 1 |
| IL.8 | D/B | 1.082418 | -0.20515 | 2.369981 | 0.594911 |
| IL.8 | D/C | 1.106919 | -0.18064 | 2.394482 | 0.491408 |
| IL17RB | A/E | -0.3459 | -1.63346 | 0.941665 | 1 |
| IL17RB | B/E | -0.32447 | -1.61203 | 0.963093 | 1 |
| IL17RB | C/E | -0.47892 | -1.76649 | 0.808641 | 1 |
| IL17RB | D/E | -0.27818 | -1.56574 | 1.009386 | 1 |
| IL17RB | B/A | 0.021428 | -1.26614 | 1.308991 | 1 |
| IL17RB | C/A | -0.13302 | -1.42059 | 1.15454 | 1 |
| IL17RB | D/A | 0.067721 | -1.21984 | 1.355285 | 1 |
| IL17RB | C/B | -0.15445 | -1.44201 | 1.133112 | 1 |
| IL17RB | D/B | 0.046294 | -1.24127 | 1.333857 | 1 |
| IL17RB | D/C | 0.200745 | -1.08682 | 1.488308 | 1 |
| IL2RA | A/E | -0.11799 | -1.40555 | 1.169577 | 1 |
| IL2RA | B/E | -0.06306 | -1.35063 | 1.2245 | 1 |
| IL2RA | C/E | -0.12032 | -1.40789 | 1.167241 | 1 |
| IL2RA | D/E | -0.00278 | -1.29034 | 1.284785 | 1 |
| IL2RA | B/A | 0.054923 | -1.23264 | 1.342486 | 1 |
| IL2RA | C/A | -0.00234 | -1.2899 | 1.285227 | 1 |
| IL2RA | D/A | 0.115208 | -1.17236 | 1.402771 | 1 |
| IL2RA | C/B | -0.05726 | -1.34482 | 1.230304 | 1 |
| IL2RA | D/B | 0.060285 | -1.22728 | 1.347848 | 1 |
| IL2RA | D/C | 0.117544 | -1.17002 | 1.405108 | 1 |
| IL6RA | A/E | -0.33998 | -1.62754 | 0.947587 | 1 |
| IL6RA | B/E | -0.33233 | -1.61989 | 0.955234 | 1 |
| IL6RA | C/E | -0.49598 | -1.78354 | 0.791583 | 1 |
| IL6RA | D/E | 0.152035 | -1.13553 | 1.439598 | 1 |
| IL6RA | B/A | 0.007647 | -1.27992 | 1.29521 | 1 |
| IL6RA | C/A | -0.156 | -1.44357 | 1.131559 | 1 |
| IL6RA | D/A | 0.492011 | -0.79555 | 1.779574 | 1 |
| IL6RA | C/B | -0.16365 | -1.45121 | 1.123912 | 1 |
| IL6RA | D/B | 0.484364 | -0.8032 | 1.771928 | 1 |
| IL6RA | D/C | 0.648016 | -0.63955 | 1.935579 | 1 |
| Kallikrein.11 | A/E | -0.44654 | -1.7341 | 0.841022 | 1 |
| Kallikrein.11 | B/E | -0.31073 | -1.5983 | 0.97683 | 1 |
| Kallikrein.11 | C/E | -0.50939 | -1.79696 | 0.77817 | 1 |
| Kallikrein.11 | D/E | -0.3087 | -1.59627 | 0.978861 | 1 |
| Kallikrein.11 | B/A | 0.135808 | -1.15176 | 1.423371 | 1 |
| Kallikrein.11 | C/A | -0.06285 | -1.35042 | 1.224711 | 1 |
| Kallikrein.11 | D/A | 0.137839 | -1.14972 | 1.425403 | 1 |
| Kallikrein.11 | C/B | -0.19866 | -1.48622 | 1.088903 | 1 |
| Kallikrein.11 | D/B | 0.002031 | -1.28553 | 1.289595 | 1 |
| Kallikrein.11 | D/C | 0.200692 | -1.08687 | 1.488255 | 1 |
| Kallikrein.6 | A/E | -0.57193 | -1.85949 | 0.715635 | 1 |
| Kallikrein.6 | B/E | -0.23184 | -1.51941 | 1.055719 | 1 |
| Kallikrein.6 | C/E | -0.24759 | -1.53516 | 1.039971 | 1 |
| Kallikrein.6 | D/E | -0.10984 | -1.3974 | 1.177724 | 1 |
| Kallikrein.6 | B/A | 0.340084 | -0.94748 | 1.627648 | 1 |
| Kallikrein.6 | C/A | 0.324337 | -0.96323 | 1.6119 | 1 |
| Kallikrein.6 | D/A | 0.46209 | -0.82547 | 1.749653 | 1 |
| Kallikrein.6 | C/B | -0.01575 | -1.30331 | 1.271816 | 1 |
| Kallikrein.6 | D/B | 0.122005 | -1.16556 | 1.409569 | 1 |
| Kallikrein.6 | D/C | 0.137753 | -1.14981 | 1.425316 | 1 |
| LAP.TGF.beta.1 | A/E | -0.38507 | -1.67264 | 0.902491 | 1 |
| LAP.TGF.beta.1 | B/E | -0.07898 | -1.36654 | 1.208587 | 1 |
| LAP.TGF.beta.1 | C/E | -0.24775 | -1.53532 | 1.039809 | 1 |
| LAP.TGF.beta.1 | D/E | 0.264267 | -1.0233 | 1.551831 | 1 |
| LAP.TGF.beta.1 | B/A | 0.306097 | -0.98147 | 1.59366 | 1 |
| LAP.TGF.beta.1 | C/A | 0.137319 | -1.15024 | 1.424882 | 1 |
| LAP.TGF.beta.1 | D/A | 0.64934 | -0.63822 | 1.936903 | 1 |
| LAP.TGF.beta.1 | C/B | -0.16878 | -1.45634 | 1.118786 | 1 |
| LAP.TGF.beta.1 | D/B | 0.343243 | -0.94432 | 1.630807 | 1 |
| LAP.TGF.beta.1 | D/C | 0.512021 | -0.77554 | 1.799585 | 1 |
| MCP.1 | A/E | -0.38498 | -1.67254 | 0.902586 | 1 |
| MCP.1 | B/E | -0.07715 | -1.36471 | 1.210416 | 1 |
| MCP.1 | C/E | -0.25963 | -1.54719 | 1.027937 | 1 |
| MCP.1 | D/E | 0.341067 | -0.9465 | 1.62863 | 1 |
| MCP.1 | B/A | 0.30783 | -0.97973 | 1.595393 | 1 |
| MCP.1 | C/A | 0.12535 | -1.16221 | 1.412914 | 1 |
| MCP.1 | D/A | 0.726044 | -0.56152 | 2.013607 | 1 |
| MCP.1 | C/B | -0.18248 | -1.47004 | 1.105084 | 1 |
| MCP.1 | D/B | 0.418214 | -0.86935 | 1.705778 | 1 |
| MCP.1 | D/C | 0.600694 | -0.68687 | 1.888257 | 1 |
| MIA | A/E | -0.63378 | -1.92134 | 0.653783 | 1 |
| MIA | B/E | -0.64632 | -1.93388 | 0.641247 | 1 |
| MIA | C/E | -0.603 | -1.89056 | 0.684566 | 1 |
| MIA | D/E | -0.2945 | -1.58207 | 0.99306 | 1 |
| MIA | B/A | -0.01254 | -1.3001 | 1.275027 | 1 |
| MIA | C/A | 0.030784 | -1.25678 | 1.318347 | 1 |
| MIA | D/A | 0.339277 | -0.94829 | 1.62684 | 1 |
| MIA | C/B | 0.04332 | -1.24424 | 1.330883 | 1 |
| MIA | D/B | 0.351813 | -0.93575 | 1.639376 | 1 |
| MIA | D/C | 0.308493 | -0.97907 | 1.596057 | 1 |
| MIC.A | A/E | -0.34743 | -1.63499 | 0.940134 | 1 |
| MIC.A | B/E | 0.068764 | -1.2188 | 1.356327 | 1 |
| MIC.A | C/E | -0.82032 | -2.10788 | 0.467242 | 0.999975 |
| MIC.A | D/E | 0.200954 | -1.08661 | 1.488517 | 1 |
| MIC.A | B/A | 0.416193 | -0.87137 | 1.703757 | 1 |
| MIC.A | C/A | -0.47289 | -1.76046 | 0.814671 | 1 |
| MIC.A | D/A | 0.548383 | -0.73918 | 1.835947 | 1 |
| MIC.A | C/B | -0.88909 | -2.17665 | 0.398478 | 0.997745 |
| MIC.A | D/B | 0.13219 | -1.15537 | 1.419754 | 1 |
| MIC.A | D/C | 1.021275 | -0.26629 | 2.308839 | 0.829279 |
| Midkine | A/E | 0.098438 | -1.18913 | 1.386001 | 1 |
| Midkine | B/E | 0.714716 | -0.57285 | 2.00228 | 1 |
| Midkine | C/E | 0.164197 | -1.12337 | 1.451761 | 1 |
| Midkine | D/E | 0.399948 | -0.88762 | 1.687511 | 1 |
| Midkine | B/A | 0.616278 | -0.67129 | 1.903842 | 1 |
| Midkine | C/A | 0.065759 | -1.2218 | 1.353323 | 1 |
| Midkine | D/A | 0.30151 | -0.98605 | 1.589073 | 1 |
| Midkine | C/B | -0.55052 | -1.83808 | 0.737044 | 1 |
| Midkine | D/B | -0.31477 | -1.60233 | 0.972795 | 1 |
| Midkine | D/C | 0.235751 | -1.05181 | 1.523314 | 1 |
| MMP.3 | A/E | -0.05737 | -1.34493 | 1.230195 | 1 |
| MMP.3 | B/E | -0.04233 | -1.32989 | 1.245236 | 1 |
| MMP.3 | C/E | -0.04818 | -1.33574 | 1.239386 | 1 |
| MMP.3 | D/E | 0.079491 | -1.20807 | 1.367054 | 1 |
| MMP.3 | B/A | 0.015041 | -1.27252 | 1.302604 | 1 |
| MMP.3 | C/A | 0.009191 | -1.27837 | 1.296754 | 1 |
| MMP.3 | D/A | 0.136859 | -1.1507 | 1.424422 | 1 |
| MMP.3 | C/B | -0.00585 | -1.29341 | 1.281713 | 1 |
| MMP.3 | D/B | 0.121818 | -1.16575 | 1.409382 | 1 |
| MMP.3 | D/C | 0.127668 | -1.1599 | 1.415232 | 1 |
| MPO | A/E | -0.09423 | -1.38179 | 1.193336 | 1 |
| MPO | B/E | 0.001222 | -1.28634 | 1.288785 | 1 |
| MPO | C/E | -0.17332 | -1.46089 | 1.114242 | 1 |
| MPO | D/E | 0.184051 | -1.10351 | 1.471614 | 1 |
| MPO | B/A | 0.09545 | -1.19211 | 1.383013 | 1 |
| MPO | C/A | -0.07909 | -1.36666 | 1.20847 | 1 |
| MPO | D/A | 0.278278 | -1.00929 | 1.565842 | 1 |
| MPO | C/B | -0.17454 | -1.46211 | 1.11302 | 1 |
| MPO | D/B | 0.182829 | -1.10473 | 1.470392 | 1 |
| MPO | D/C | 0.357372 | -0.93019 | 1.644936 | 1 |
| MYD88 | A/E | -0.40192 | -1.68948 | 0.885647 | 1 |
| MYD88 | B/E | 0.000381 | -1.28718 | 1.287945 | 1 |
| MYD88 | C/E | -0.24244 | -1.53 | 1.045125 | 1 |
| MYD88 | D/E | -0.14605 | -1.43361 | 1.141514 | 1 |
| MYD88 | B/A | 0.402298 | -0.88527 | 1.689862 | 1 |
| MYD88 | C/A | 0.159479 | -1.12808 | 1.447042 | 1 |
| MYD88 | D/A | 0.255868 | -1.0317 | 1.543431 | 1 |
| MYD88 | C/B | -0.24282 | -1.53038 | 1.044744 | 1 |
| MYD88 | D/B | -0.14643 | -1.43399 | 1.141133 | 1 |
| MYD88 | D/C | 0.096389 | -1.19117 | 1.383953 | 1 |
| Osteoprotegerin | A/E | -0.32379 | -1.61136 | 0.963771 | 1 |
| Osteoprotegerin | B/E | -0.02155 | -1.30911 | 1.266012 | 1 |
| Osteoprotegerin | C/E | -0.27586 | -1.56342 | 1.011706 | 1 |
| Osteoprotegerin | D/E | -0.02826 | -1.31583 | 1.259302 | 1 |
| Osteoprotegerin | B/A | 0.302241 | -0.98532 | 1.589805 | 1 |
| Osteoprotegerin | C/A | 0.047936 | -1.23963 | 1.335499 | 1 |
| Osteoprotegerin | D/A | 0.295531 | -0.99203 | 1.583094 | 1 |
| Osteoprotegerin | C/B | -0.25431 | -1.54187 | 1.033258 | 1 |
| Osteoprotegerin | D/B | -0.00671 | -1.29427 | 1.280853 | 1 |
| Osteoprotegerin | D/C | 0.247595 | -1.03997 | 1.535159 | 1 |
| PDGF.subunit.B | A/E | -0.73187 | -2.01943 | 0.555694 | 1 |
| PDGF.subunit.B | B/E | -0.14729 | -1.43485 | 1.140275 | 1 |
| PDGF.subunit.B | C/E | -0.23398 | -1.52154 | 1.053586 | 1 |
| PDGF.subunit.B | D/E | 0.916642 | -0.37092 | 2.204206 | 0.991819 |
| PDGF.subunit.B | B/A | 0.584581 | -0.70298 | 1.872144 | 1 |
| PDGF.subunit.B | C/A | 0.497891 | -0.78967 | 1.785455 | 1 |
| PDGF.subunit.B | D/A | 1.648511 | 0.360948 | 2.936075 | 3.02E-05 |
| PDGF.subunit.B | C/B | -0.08669 | -1.37425 | 1.200874 | 1 |
| PDGF.subunit.B | D/B | 1.06393 | -0.22363 | 2.351494 | 0.672233 |
| PDGF.subunit.B | D/C | 1.15062 | -0.13694 | 2.438184 | 0.322841 |
| PECAM.1 | A/E | -0.48737 | -1.77494 | 0.800192 | 1 |
| PECAM.1 | B/E | -0.25059 | -1.53815 | 1.036972 | 1 |
| PECAM.1 | C/E | -0.4692 | -1.75676 | 0.818366 | 1 |
| PECAM.1 | D/E | -0.15511 | -1.44267 | 1.132458 | 1 |
| PECAM.1 | B/A | 0.236781 | -1.05078 | 1.524344 | 1 |
| PECAM.1 | C/A | 0.018174 | -1.26939 | 1.305738 | 1 |
| PECAM.1 | D/A | 0.332266 | -0.9553 | 1.619829 | 1 |
| PECAM.1 | C/B | -0.21861 | -1.50617 | 1.068957 | 1 |
| PECAM.1 | D/B | 0.095485 | -1.19208 | 1.383049 | 1 |
| PECAM.1 | D/C | 0.314092 | -0.97347 | 1.601655 | 1 |
| PlGF | A/E | -0.64893 | -1.93649 | 0.638636 | 1 |
| PlGF | B/E | -0.36402 | -1.65158 | 0.923547 | 1 |
| PlGF | C/E | -0.56469 | -1.85226 | 0.72287 | 1 |
| PlGF | D/E | -0.31558 | -1.60314 | 0.971987 | 1 |
| PlGF | B/A | 0.284912 | -1.00265 | 1.572475 | 1 |
| PlGF | C/A | 0.084234 | -1.20333 | 1.371798 | 1 |
| PlGF | D/A | 0.333351 | -0.95421 | 1.620914 | 1 |
| PlGF | C/B | -0.20068 | -1.48824 | 1.086886 | 1 |
| PlGF | D/B | 0.048439 | -1.23912 | 1.336003 | 1 |
| PlGF | D/C | 0.249117 | -1.03845 | 1.53668 | 1 |
| Prolactin | A/E | 1.228535 | -0.05903 | 2.516098 | 0.122194 |
| Prolactin | B/E | 0.975752 | -0.31181 | 2.263315 | 0.938496 |
| Prolactin | C/E | 1.650283 | 0.36272 | 2.937847 | 2.89E-05 |
| Prolactin | D/E | -0.03449 | -1.32206 | 1.253071 | 1 |
| Prolactin | B/A | -0.25278 | -1.54035 | 1.03478 | 1 |
| Prolactin | C/A | 0.421749 | -0.86581 | 1.709312 | 1 |
| Prolactin | D/A | -1.26303 | -2.55059 | 0.024536 | 0.073555 |
| Prolactin | C/B | 0.674532 | -0.61303 | 1.962095 | 1 |
| Prolactin | D/B | -1.01024 | -2.29781 | 0.277319 | 0.862 |
| Prolactin | D/C | -1.68478 | -2.97234 | -0.39721 | 1.24E-05 |
| Prostasin | A/E | -0.09506 | -1.38262 | 1.192506 | 1 |
| Prostasin | B/E | -0.0761 | -1.36367 | 1.211462 | 1 |
| Prostasin | C/E | -0.21866 | -1.50622 | 1.068907 | 1 |
| Prostasin | D/E | 0.081616 | -1.20595 | 1.36918 | 1 |
| Prostasin | B/A | 0.018956 | -1.26861 | 1.306519 | 1 |
| Prostasin | C/A | -0.1236 | -1.41116 | 1.163964 | 1 |
| Prostasin | D/A | 0.176673 | -1.11089 | 1.464237 | 1 |
| Prostasin | C/B | -0.14256 | -1.43012 | 1.145008 | 1 |
| Prostasin | D/B | 0.157718 | -1.12985 | 1.445281 | 1 |
| Prostasin | D/C | 0.300273 | -0.98729 | 1.587836 | 1 |
| PSA | A/E | 0.730205 | -0.55736 | 2.017768 | 1 |
| PSA | B/E | -0.24568 | -1.53324 | 1.041882 | 1 |
| PSA | C/E | -0.34369 | -1.63125 | 0.943872 | 1 |
| PSA | D/E | 0.125083 | -1.16248 | 1.412646 | 1 |
| PSA | B/A | -0.97589 | -2.26345 | 0.311677 | 0.938273 |
| PSA | C/A | -1.0739 | -2.36146 | 0.213667 | 0.630876 |
| PSA | D/A | -0.60512 | -1.89269 | 0.682441 | 1 |
| PSA | C/B | -0.09801 | -1.38557 | 1.189553 | 1 |
| PSA | D/B | 0.370764 | -0.9168 | 1.658327 | 1 |
| PSA | D/C | 0.468774 | -0.81879 | 1.756337 | 1 |
| REG.4 | A/E | -0.01744 | -1.30501 | 1.27012 | 1 |
| REG.4 | B/E | -0.0483 | -1.33586 | 1.239265 | 1 |
| REG.4 | C/E | -0.1459 | -1.43346 | 1.141667 | 1 |
| REG.4 | D/E | 0.288647 | -0.99892 | 1.57621 | 1 |
| REG.4 | B/A | -0.03085 | -1.31842 | 1.256709 | 1 |
| REG.4 | C/A | -0.12845 | -1.41602 | 1.15911 | 1 |
| REG.4 | D/A | 0.30609 | -0.98147 | 1.593654 | 1 |
| REG.4 | C/B | -0.0976 | -1.38516 | 1.189965 | 1 |
| REG.4 | D/B | 0.336945 | -0.95062 | 1.624508 | 1 |
| REG.4 | D/C | 0.434544 | -0.85302 | 1.722107 | 1 |
| Stem.cell.factor | A/E | -0.25866 | -1.54622 | 1.028906 | 1 |
| Stem.cell.factor | B/E | -0.26823 | -1.55579 | 1.019333 | 1 |
| Stem.cell.factor | C/E | -0.44963 | -1.73719 | 0.837933 | 1 |
| Stem.cell.factor | D/E | -0.40021 | -1.68778 | 0.887349 | 1 |
| Stem.cell.factor | B/A | -0.00957 | -1.29714 | 1.27799 | 1 |
| Stem.cell.factor | C/A | -0.19097 | -1.47854 | 1.09659 | 1 |
| Stem.cell.factor | D/A | -0.14156 | -1.42912 | 1.146006 | 1 |
| Stem.cell.factor | C/B | -0.1814 | -1.46896 | 1.106163 | 1 |
| Stem.cell.factor | D/B | -0.13198 | -1.41955 | 1.155579 | 1 |
| Stem.cell.factor | D/C | 0.049416 | -1.23815 | 1.336979 | 1 |
| TGF.alpha | A/E | 0.01454 | -1.27302 | 1.302104 | 1 |
| TGF.alpha | B/E | -0.05146 | -1.33903 | 1.2361 | 1 |
| TGF.alpha | C/E | -0.2037 | -1.49126 | 1.083867 | 1 |
| TGF.alpha | D/E | -0.16417 | -1.45173 | 1.123398 | 1 |
| TGF.alpha | B/A | -0.066 | -1.35357 | 1.22156 | 1 |
| TGF.alpha | C/A | -0.21824 | -1.5058 | 1.069327 | 1 |
| TGF.alpha | D/A | -0.17871 | -1.46627 | 1.108857 | 1 |
| TGF.alpha | C/B | -0.15223 | -1.4398 | 1.13533 | 1 |
| TGF.alpha | D/B | -0.1127 | -1.40027 | 1.174861 | 1 |
| TGF.alpha | D/C | 0.039531 | -1.24803 | 1.327094 | 1 |
| Thrombopoietin | A/E | -0.37393 | -1.66149 | 0.913635 | 1 |
| Thrombopoietin | B/E | -0.14577 | -1.43334 | 1.141791 | 1 |
| Thrombopoietin | C/E | -0.33228 | -1.61984 | 0.955288 | 1 |
| Thrombopoietin | D/E | -0.11724 | -1.4048 | 1.170322 | 1 |
| Thrombopoietin | B/A | 0.228157 | -1.05941 | 1.51572 | 1 |
| Thrombopoietin | C/A | 0.041654 | -1.24591 | 1.329217 | 1 |
| Thrombopoietin | D/A | 0.256687 | -1.03088 | 1.544251 | 1 |
| Thrombopoietin | C/B | -0.1865 | -1.47407 | 1.10106 | 1 |
| Thrombopoietin | D/B | 0.028531 | -1.25903 | 1.316094 | 1 |
| Thrombopoietin | D/C | 0.215034 | -1.07253 | 1.502597 | 1 |
| TIE2 | A/E | -0.22035 | -1.50791 | 1.067217 | 1 |
| TIE2 | B/E | -0.22496 | -1.51252 | 1.062603 | 1 |
| TIE2 | C/E | -0.22292 | -1.51048 | 1.064642 | 1 |
| TIE2 | D/E | 0.048792 | -1.23877 | 1.336356 | 1 |
| TIE2 | B/A | -0.00461 | -1.29218 | 1.282949 | 1 |
| TIE2 | C/A | -0.00257 | -1.29014 | 1.284989 | 1 |
| TIE2 | D/A | 0.269139 | -1.01842 | 1.556702 | 1 |
| TIE2 | C/B | 0.002039 | -1.28552 | 1.289603 | 1 |
| TIE2 | D/B | 0.273753 | -1.01381 | 1.561316 | 1 |
| TIE2 | D/C | 0.271714 | -1.01585 | 1.559277 | 1 |
| Tissue.Factor | A/E | -0.39283 | -1.68039 | 0.894736 | 1 |
| Tissue.Factor | B/E | -0.24589 | -1.53345 | 1.041678 | 1 |
| Tissue.Factor | C/E | -0.44157 | -1.72913 | 0.845997 | 1 |
| Tissue.Factor | D/E | -0.44497 | -1.73254 | 0.842591 | 1 |
| Tissue.Factor | B/A | 0.146941 | -1.14062 | 1.434505 | 1 |
| Tissue.Factor | C/A | -0.04874 | -1.3363 | 1.238824 | 1 |
| Tissue.Factor | D/A | -0.05215 | -1.33971 | 1.235418 | 1 |
| Tissue.Factor | C/B | -0.19568 | -1.48324 | 1.091882 | 1 |
| Tissue.Factor | D/B | -0.19909 | -1.48665 | 1.088477 | 1 |
| Tissue.Factor | D/C | -0.00341 | -1.29097 | 1.284158 | 1 |
| TNF | A/E | -0.00529 | -1.29286 | 1.28227 | 1 |
| TNF | B/E | -0.03084 | -1.31841 | 1.25672 | 1 |
| TNF | C/E | 0.068782 | -1.21878 | 1.356346 | 1 |
| TNF | D/E | 0.112691 | -1.17487 | 1.400255 | 1 |
| TNF | B/A | -0.02555 | -1.31311 | 1.262014 | 1 |
| TNF | C/A | 0.074076 | -1.21349 | 1.361639 | 1 |
| TNF | D/A | 0.117985 | -1.16958 | 1.405548 | 1 |
| TNF | C/B | 0.099626 | -1.18794 | 1.387189 | 1 |
| TNF | D/B | 0.143535 | -1.14403 | 1.431098 | 1 |
| TNF | D/C | 0.043909 | -1.24365 | 1.331472 | 1 |
| TNF.R2 | A/E | -0.41884 | -1.70641 | 0.868721 | 1 |
| TNF.R2 | B/E | -0.0639 | -1.35146 | 1.223665 | 1 |
| TNF.R2 | C/E | -0.3718 | -1.65936 | 0.915762 | 1 |
| TNF.R2 | D/E | 0.006443 | -1.28112 | 1.294006 | 1 |
| TNF.R2 | B/A | 0.354944 | -0.93262 | 1.642507 | 1 |
| TNF.R2 | C/A | 0.047041 | -1.24052 | 1.334605 | 1 |
| TNF.R2 | D/A | 0.425286 | -0.86228 | 1.712849 | 1 |
| TNF.R2 | C/B | -0.3079 | -1.59547 | 0.979661 | 1 |
| TNF.R2 | D/B | 0.070342 | -1.21722 | 1.357905 | 1 |
| TNF.R2 | D/C | 0.378244 | -0.90932 | 1.665808 | 1 |
| TNF.RI | A/E | -0.27892 | -1.56648 | 1.008647 | 1 |
| TNF.RI | B/E | 0.016103 | -1.27146 | 1.303666 | 1 |
| TNF.RI | C/E | -0.18978 | -1.47734 | 1.097785 | 1 |
| TNF.RI | D/E | -0.06358 | -1.35114 | 1.223986 | 1 |
| TNF.RI | B/A | 0.29502 | -0.99254 | 1.582583 | 1 |
| TNF.RI | C/A | 0.089138 | -1.19843 | 1.376702 | 1 |
| TNF.RI | D/A | 0.21534 | -1.07222 | 1.502903 | 1 |
| TNF.RI | C/B | -0.20588 | -1.49344 | 1.081682 | 1 |
| TNF.RI | D/B | -0.07968 | -1.36724 | 1.207883 | 1 |
| TNF.RI | D/C | 0.126201 | -1.16136 | 1.413765 | 1 |
| TNFRSF4 | A/E | -0.45869 | -1.74625 | 0.828874 | 1 |
| TNFRSF4 | B/E | -0.0136 | -1.30116 | 1.273963 | 1 |
| TNFRSF4 | C/E | -0.20048 | -1.48805 | 1.087082 | 1 |
| TNFRSF4 | D/E | -0.05732 | -1.34488 | 1.230248 | 1 |
| TNFRSF4 | B/A | 0.445089 | -0.84247 | 1.732652 | 1 |
| TNFRSF4 | C/A | 0.258208 | -1.02936 | 1.545771 | 1 |
| TNFRSF4 | D/A | 0.401374 | -0.88619 | 1.688937 | 1 |
| TNFRSF4 | C/B | -0.18688 | -1.47444 | 1.100682 | 1 |
| TNFRSF4 | D/B | -0.04371 | -1.33128 | 1.243849 | 1 |
| TNFRSF4 | D/C | 0.143166 | -1.1444 | 1.43073 | 1 |
| TNFSF14 | A/E | 0.040668 | -1.2469 | 1.328231 | 1 |
| TNFSF14 | B/E | 0.299366 | -0.9882 | 1.58693 | 1 |
| TNFSF14 | C/E | 0.009446 | -1.27812 | 1.297009 | 1 |
| TNFSF14 | D/E | 0.276483 | -1.01108 | 1.564047 | 1 |
| TNFSF14 | B/A | 0.258699 | -1.02886 | 1.546262 | 1 |
| TNFSF14 | C/A | -0.03122 | -1.31879 | 1.256341 | 1 |
| TNFSF14 | D/A | 0.235815 | -1.05175 | 1.523379 | 1 |
| TNFSF14 | C/B | -0.28992 | -1.57748 | 0.997643 | 1 |
| TNFSF14 | D/B | -0.02288 | -1.31045 | 1.26468 | 1 |
| TNFSF14 | D/C | 0.267037 | -1.02053 | 1.554601 | 1 |
| TR.AP | A/E | -0.35322 | -1.64079 | 0.934339 | 1 |
| TR.AP | B/E | -0.19405 | -1.48161 | 1.093517 | 1 |
| TR.AP | C/E | -0.28759 | -1.57516 | 0.999971 | 1 |
| TR.AP | D/E | 0.055106 | -1.23246 | 1.34267 | 1 |
| TR.AP | B/A | 0.159178 | -1.12839 | 1.446742 | 1 |
| TR.AP | C/A | 0.065632 | -1.22193 | 1.353196 | 1 |
| TR.AP | D/A | 0.408331 | -0.87923 | 1.695894 | 1 |
| TR.AP | C/B | -0.09355 | -1.38111 | 1.194017 | 1 |
| TR.AP | D/B | 0.249153 | -1.03841 | 1.536716 | 1 |
| TR.AP | D/C | 0.342699 | -0.94486 | 1.630262 | 1 |
| U.PAR | A/E | -0.11941 | -1.40697 | 1.168158 | 1 |
| U.PAR | B/E | -0.00132 | -1.28888 | 1.286246 | 1 |
| U.PAR | C/E | -0.26198 | -1.54954 | 1.025584 | 1 |
| U.PAR | D/E | 0.0469 | -1.24066 | 1.334463 | 1 |
| U.PAR | B/A | 0.118089 | -1.16947 | 1.405652 | 1 |
| U.PAR | C/A | -0.14257 | -1.43014 | 1.14499 | 1 |
| U.PAR | D/A | 0.166306 | -1.12126 | 1.453869 | 1 |
| U.PAR | C/B | -0.26066 | -1.54823 | 1.026902 | 1 |
| U.PAR | D/B | 0.048217 | -1.23935 | 1.33578 | 1 |
| U.PAR | D/C | 0.308879 | -0.97868 | 1.596442 | 1 |
| VEGF.A | A/E | -0.38499 | -1.67255 | 0.902576 | 1 |
| VEGF.A | B/E | -0.01992 | -1.30749 | 1.26764 | 1 |
| VEGF.A | C/E | -0.24658 | -1.53414 | 1.040988 | 1 |
| VEGF.A | D/E | 0.092884 | -1.19468 | 1.380447 | 1 |
| VEGF.A | B/A | 0.365065 | -0.9225 | 1.652628 | 1 |
| VEGF.A | C/A | 0.138413 | -1.14915 | 1.425976 | 1 |
| VEGF.A | D/A | 0.477872 | -0.80969 | 1.765435 | 1 |
| VEGF.A | C/B | -0.22665 | -1.51422 | 1.060912 | 1 |
| VEGF.A | D/B | 0.112807 | -1.17476 | 1.40037 | 1 |
| VEGF.A | D/C | 0.339459 | -0.9481 | 1.627022 | 1 |
| VEGF.D | A/E | -0.16086 | -1.44842 | 1.126708 | 1 |
| VEGF.D | B/E | -0.23253 | -1.52009 | 1.055032 | 1 |
| VEGF.D | C/E | -0.17385 | -1.46142 | 1.11371 | 1 |
| VEGF.D | D/E | -0.1297 | -1.41726 | 1.157868 | 1 |
| VEGF.D | B/A | -0.07168 | -1.35924 | 1.215887 | 1 |
| VEGF.D | C/A | -0.013 | -1.30056 | 1.274565 | 1 |
| VEGF.D | D/A | 0.03116 | -1.2564 | 1.318723 | 1 |
| VEGF.D | C/B | 0.058678 | -1.22889 | 1.346242 | 1 |
| VEGF.D | D/B | 0.102836 | -1.18473 | 1.390399 | 1 |
| VEGF.D | D/C | 0.044158 | -1.24341 | 1.331721 | 1 |
| VEGFR.2 | A/E | -0.15969 | -1.44725 | 1.127876 | 1 |
| VEGFR.2 | B/E | -0.26163 | -1.5492 | 1.02593 | 1 |
| VEGFR.2 | C/E | -0.24783 | -1.53539 | 1.039737 | 1 |
| VEGFR.2 | D/E | -0.15742 | -1.44499 | 1.13014 | 1 |
| VEGFR.2 | B/A | -0.10195 | -1.38951 | 1.185617 | 1 |
| VEGFR.2 | C/A | -0.08814 | -1.3757 | 1.199424 | 1 |
| VEGFR.2 | D/A | 0.002264 | -1.2853 | 1.289827 | 1 |
| VEGFR.2 | C/B | 0.013807 | -1.27376 | 1.301371 | 1 |
| VEGFR.2 | D/B | 0.10421 | -1.18335 | 1.391773 | 1 |
| VEGFR.2 | D/C | 0.090403 | -1.19716 | 1.377966 | 1 |

| **Table S4: Anova Table (Type II tests) or 2-Way Anova factor analysis for Proseek Assay** | | | | | |
| --- | --- | --- | --- | --- | --- |
|  | **SS** | **Df** | **F-value** | **Pr(>F)** | **Sig** |
| Group | 73 | 2 | 6.49E+01 | 2.20E-16 | *** |
| Proteins | 65377 | 91 | 1.28E+03 | 2.20E-16 | *** |
| Sample | 766 | 72 | 1.89E+01 | 2.20E-16 | *** |
| Group:Proteins | 228 | 182 | 2.23E+00 | 2.20E-16 | *** |
| Residuals | 3942 | 7012 |  |  |  |
| Sig Codes: 0 ‘***’, 0.001 ‘**’, 0.01 ‘*’   \| **Comparison** \| \| **Protein** \| **Difference** \| **Lower CI** \| **Upper CI** \| **Q-value** \| \| --- \| --- \| --- \| --- \| --- \| --- \| --- \| \| Malignant \| Benign \| CEA \| 1.97 \| 1.08 \| 2.85 \| 0.00 \| \| Malignant \| Healthy \| CEA \| 2.11 \| 1.02 \| 3.19 \| 0.00 \| \| Malignant \| Healthy \| IL.8 \| 1.22 \| 0.14 \| 2.31 \| 0.00 \| \| Benign \| Healthy \| Prolactin \| 1.10 \| 0.02 \| 2.19 \| 0.04 \| \| Malignant \| Benign \| IL.8 \| 0.81 \| -0.07 \| 1.70 \| 0.21 \| \| Malignant \| Benign \| PDGF.subunit.B \| 0.78 \| -0.10 \| 1.67 \| 0.34 \| \| Malignant \| Healthy \| Prolactin \| 0.81 \| -0.28 \| 1.89 \| 0.92 \| \| Malignant \| Benign \| CXCL5 \| 0.63 \| -0.26 \| 1.52 \| 0.98 \| \| Malignant \| Benign \| Amphiregulin \| 0.62 \| -0.27 \| 1.51 \| 0.99 \| | | | | | |

| **Table S5: p- values calculated for stage specific protein expressions analyzed by Bio-Plex Assay (Stage specific (A-D, n=15) and healthy group E** | | | | | | | | | | | |
| --- | --- | --- | --- | --- | --- | --- | --- | --- | --- | --- | --- |
| **Comparison** | **Target Biomarker** | **p-adju** | **Comparison** | **Target Biomarker** | **p-adju** | **Comparison** | **Target Biomarker** | **p-adju** | **Comparison** | **Target Biomarker** | **p-adju** |
| A/E | Hu.IL.8..54. | 0.006 | B/E | Hu.IL.8..54. | 0.002 | C/E | Hu.MIP.1b..18. | 0.017 | D/E | Hu.PDGF.bb..47. | 2E-07 |
| A/E | Hu.MCP.1.MCAF...53. | 0.053 | B/E | Hu.MCP.1.MCAF...53. | 0.074 | C/E | Hu.IL.8..54. | 0.066 | D/E | Hu.IL.8..54. | 6E-05 |
| A/E | Hu.IL.4..52. | 0.113 | B/E | Hu.IL.4..52. | 0.210 | C/E | Hu.IL.4..52. | 0.215 | D/E | Hu.MCP.1.MCAF...53. | 5E-04 |
| A/E | Hu.G.CSF..57. | 0.275 | B/E | Hu.IL.6..19. | 0.223 | C/E | Hu.IL.15..73. | 0.376 | D/E | Hu.IFN.g..21. | 2E-03 |
| A/E | Hu.MIP.1b..18. | 0.522 | B/E | Hu.PDGF.bb..47. | 0.739 | C/E | Hu.IL.2..38. | 0.712 | D/E | Hu.IL.4..52. | 4E-03 |
| A/E | Hu.IL.7..74. | 0.954 | B/E | Hu.G.CSF..57. | 0.741 | C/E | Hu.PDGF.bb..47. | 0.744 | D/A | Hu.PDGF.bb..47. | 4E-03 |
| A/E | Hu.IL.6..19. | 0.978 | B/E | Hu.IL.9..77. | 1.000 | C/E | Hu.G.CSF..57. | 0.757 | D/E | Hu.MIP.1a..55. | 4E-03 |
| A/E | Hu.IL.5..33. | 0.986 | B/E | Hu.VEGF..45. | 1.000 | C/E | Hu.GM.CSF..34. | 0.796 | D/E | Hu.IL.1b..39. | 1E-02 |
| A/E | Hu.VEGF..45. | 0.992 | B/E | Hu.IL.7..74. | 1.000 | C/E | Hu.MCP.1.MCAF...53. | 0.887 | D/E | Hu.IL.9..77. | 2E-02 |
| A/E | Hu.IL.13..51. | 0.995 | B/E | Hu.GM.CSF..34. | 1.000 | C/E | Hu.IL.9..77. | 0.987 | D/E | Hu.IL.6..19. | 3E-02 |
| A/E | Hu.IFN.g..21. | 1.000 | B/E | Hu.IFN.g..21. | 1.000 | C/E | Hu.IL.6..19. | 0.993 | D/E | Hu.IL.7..74. | 6E-02 |
| A/E | Hu.IL.10..56. | 1.000 | B/E | Hu.MIP.1b..18. | 1.000 | C/E | Hu.VEGF..45. | 0.998 | D/E | Hu.IL.17..76. | 9E-02 |
| A/E | Hu.IL.15..73. | 1.000 | B/E | Hu.TNF.a..36. | 1.000 | C/E | Hu.IL.5..33. | 0.998 | D/A | Hu.MIP.1a..55. | 9E-02 |
| A/E | Hu.IL.9..77. | 1.000 | B/A | Hu.IL.13..51. | 1.000 | C/B | Hu.IL.2..38. | 0.999 | D/C | Hu.MIP.1a..55. | 1E-01 |
| A/E | Hu.GM.CSF..34. | 1.000 | B/A | Hu.IL.5..33. | 1.000 | C/E | Hu.IL.1ra..25. | 1.000 | D/E | Hu.G.CSF..57. | 1E-01 |
| A/E | Hu.TNF.a..36. | 1.000 | B/E | Hu.IL.10..56. | 1.000 | C/E | Hu.IFN.g..21. | 1.000 | D/E | Hu.MIP.1b..18. | 2E-01 |
| A/E | Hu.Eotaxin..43. | 1.000 | B/E | Hu.IL.1b..39. | 1.000 | C/B | Hu.MIP.1b..18. | 1.000 | D/E | Hu.TNF.a..36. | 2E-01 |
| A/E | Hu.IL.1b..39. | 1.000 | B/E | Hu.Eotaxin..43. | 1.000 | C/E | Hu.Eotaxin..43. | 1.000 | D/E | Hu.IL.5..33. | 2E-01 |
| A/E | Hu.FGF.basic..44. | 1.000 | B/A | Hu.Eotaxin..43. | 1.000 | C/E | Hu.IL.17..76. | 1.000 | D/E | Hu.VEGF..45. | 3E-01 |
| A/E | Hu.IL.12.p70...75. | 1.000 | B/A | Hu.FGF.basic..44. | 1.000 | C/A | Hu.IL.2..38. | 1.000 | D/E | Hu.FGF.basic..44. | 5E-01 |
| A/E | Hu.IL.17..76. | 1.000 | B/A | Hu.FGF.basic..44. | 1.000 | C/B | Hu.IL.15..73. | 1.000 | D/B | Hu.MIP.1a..55. | 5E-01 |
| A/E | Hu.IL.1ra..25. | 1.000 | B/A | Hu.G.CSF..57. | 1.000 | C/E | Hu.IL.7..74. | 1 | D/E | Hu.IL.1ra..25. | 6E-01 |
| A/E | Hu.IL.2..38. | 1.000 | B/A | Hu.GM.CSF..34. | 1.000 | C/E | Hu.TNF.a..36. | 1 | D/E | Hu.IL.10..56. | 8E-01 |
| A/E | Hu.IP.10..48. | 1.000 | B/A | Hu.IFN.g..21. | 1.000 | C/E | Hu.IL.10..56. | 1 | D/C | Hu.PDGF.bb..47. | 8E-01 |
| A/E | Hu.MIP.1a..55. | 1.000 | B/A | Hu.IL.10..56. | 1.000 | C/E | Hu.IL.13..51. | 1 | D/B | Hu.PDGF.bb..47. | 8E-01 |
| A/E | Hu.PDGF.bb..47. | 1.000 | B/E | Hu.IL.12.p70...75. | 1.000 | C/A | Hu.Eotaxin..43. | 1 | D/A | Hu.FGF.basic..44. | 9E-01 |
| A/E | Hu.RANTES..37. | 1.000 | B/A | Hu.IL.12.p70...75. | 1.000 | C/B | Hu.Eotaxin..43. | 1 | D/C | Hu.IL.1b..39. | 1E+00 |
|  |  |  | B/E | Hu.IL.13..51. | 1.000 | C/E | Hu.FGF.basic..44. | 1 | D/A | Hu.IL.1b..39. | 1E+00 |
|  |  |  | B/E | Hu.IL.15..73. | 1.000 | C/A | Hu.FGF.basic..44. | 1 | D/B | Hu.IL.1b..39. | 1E+00 |
|  |  |  | B/A | Hu.IL.15..73. | 1.000 | C/B | Hu.FGF.basic..44. | 1 | D/E | Hu.IL.2..38. | 1E+00 |
|  |  |  | B/E | Hu.IL.17..76. | 1.000 | C/A | Hu.G.CSF..57. | 1 | D/E | Hu.GM.CSF..34. | 1E+00 |
|  |  |  | B/A | Hu.IL.17..76. | 1.000 | C/B | Hu.G.CSF..57. | 1 | D/A | Hu.IL.17..76. | 1E+00 |
|  |  |  | B/A | Hu.IL.1b..39. | 1.000 | C/A | Hu.GM.CSF..34. | 1 | D/C | Hu.FGF.basic..44. | 1E+00 |
|  |  |  | B/E | Hu.IL.1ra..25. | 1.000 | C/B | Hu.GM.CSF..34. | 1 | D/E | Hu.IL.15..73. | 1E+00 |
|  |  |  | B/A | Hu.IL.1ra..25. | 1.000 | C/A | Hu.IFN.g..21. | 1 | D/B | Hu.IFN.g..21. | 1E+00 |
|  |  |  | B/E | Hu.IL.2..38. | 1.000 | C/B | Hu.IFN.g..21. | 1 | D/C | Hu.IFN.g..21. | 1E+00 |
|  |  |  | B/A | Hu.IL.2..38. | 1.000 | C/A | Hu.IL.10..56. | 1 | D/B | Hu.FGF.basic..44. | 1E+00 |
|  |  |  | B/A | Hu.IL.4..52. | 1.000 | C/B | Hu.IL.10..56. | 1 | D/A | Hu.IFN.g..21. | 1E+00 |
|  |  |  | B/A | Hu.IL.5..33. | 1.000 | C/E | Hu.IL.12.p70...75. | 1 | D/B | Hu.IL.17..76. | 1E+00 |
|  |  |  | B/A | Hu.IL.6..19. | 1.000 | C/A | Hu.IL.12.p70...75. | 1 | D/A | Hu.IL.9..77. | 1E+00 |
|  |  |  | B/A | Hu.IL.7..74. | 1.000 | C/B | Hu.IL.12.p70...75. | 1 | D/E | Hu.IL.12.p70...75. | 1E+00 |
|  |  |  | B/A | Hu.IL.8..54. | 1.000 | C/A | Hu.IL.13..51. | 1 | D/C | Hu.MCP.1.MCAF...53. | 1E+00 |
|  |  |  | B/A | Hu.IL.9..77. | 1.000 | C/B | Hu.IL.13..51. | 1 | D/C | Hu.IL.7..74. | 1E+00 |
|  |  |  | B/E | Hu.IP.10..48. | 1.000 | C/A | Hu.IL.15..73. | 1 | D/B | Hu.IL.1ra..25. | 1E+00 |
|  |  |  | B/A | Hu.IP.10..48. | 1.000 | C/A | Hu.IL.17..76. | 1 | D/B | Hu.IL.9..77. | 1E+00 |
|  |  |  | B/A | Hu.MCP.1.MCAF...53. | 1.000 | C/B | Hu.IL.17..76. | 1 | D/C | Hu.IL.17..76. | 1E+00 |
|  |  |  | B/E | Hu.MIP.1a..55. | 1.000 | C/E | Hu.IL.1b..39. | 1 | D/B | Hu.IL.5..33. | 1E+00 |
|  |  |  | B/A | Hu.MIP.1a..55. | 1.000 | C/A | Hu.IL.1b..39. | 1 | D/A | Hu.TNF.a..36. | 1E+00 |
|  |  |  | B/A | Hu.MIP.1b..18. | 1.000 | C/B | Hu.IL.1b..39. | 1 | D/B | Hu.IL.2..38. | 1E+00 |
|  |  |  | B/A | Hu.PDGF.bb..47. | 1.000 | C/A | Hu.IL.1ra..25. | 1 | D/C | Hu.TNF.a..36. | 1E+00 |
|  |  |  | B/E | Hu.RANTES..37. | 1.000 | C/B | Hu.IL.1ra..25. | 1 | D/B | Hu.TNF.a..36. | 1E+00 |
|  |  |  | B/A | Hu.RANTES..37. | 1.000 | C/A | Hu.IL.4..52. | 1 | D/B | Hu.MIP.1b..18. | 1E+00 |
|  |  |  | B/A | Hu.TNF.a..36. | 1.000 | C/B | Hu.IL.4..52. | 1 | D/A | Hu.IP.10..48. | 1E+00 |
|  |  |  | B/A | Hu.VEGF..45. | 1.000 | C/A | Hu.IL.5..33. | 1 | D/B | Hu.IL.7..74. | 1E+00 |
|  |  |  |  |  |  | C/B | Hu.IL.5..33. | 1 | D/E | Hu.Eotaxin..43. | 1E+00 |
|  |  |  |  |  |  | C/A | Hu.IL.6..19. | 1 | D/A | Hu.Eotaxin..43. | 1E+00 |
|  |  |  |  |  |  | C/B | Hu.IL.6..19. | 1 | D/B | Hu.Eotaxin..43. | 1E+00 |
|  |  |  |  |  |  | C/A | Hu.IL.7..74. | 1 | D/C | Hu.Eotaxin..43. | 1E+00 |
|  |  |  |  |  |  | C/B | Hu.IL.7..74. | 1 | D/A | Hu.G.CSF..57. | 1E+00 |
|  |  |  |  |  |  | C/A | Hu.IL.8..54. | 1 | D/B | Hu.G.CSF..57. | 1E+00 |
|  |  |  |  |  |  | C/B | Hu.IL.8..54. | 1 | D/C | Hu.G.CSF..57. | 1E+00 |
|  |  |  |  |  |  | C/A | Hu.IL.9..77. | 1 | D/A | Hu.GM.CSF..34. | 1E+00 |
|  |  |  |  |  |  | C/B | Hu.IL.9..77. | 1 | D/B | Hu.GM.CSF..34. | 1E+00 |
|  |  |  |  |  |  | C/E | Hu.IP.10..48. | 1 | D/C | Hu.GM.CSF..34. | 1E+00 |
|  |  |  |  |  |  | C/A | Hu.IP.10..48. | 1 | D/A | Hu.IL.10..56. | 1E+00 |
|  |  |  |  |  |  | C/B | Hu.IP.10..48. | 1 | D/B | Hu.IL.10..56. | 1E+00 |
|  |  |  |  |  |  | C/A | Hu.MCP.1.MCAF...53. | 1 | D/C | Hu.IL.10..56. | 1E+00 |
|  |  |  |  |  |  | C/B | Hu.MCP.1.MCAF...53. | 1 | D/A | Hu.IL.12.p70...75. | 1E+00 |
|  |  |  |  |  |  | C/E | Hu.MIP.1a..55. | 1 | D/B | Hu.IL.12.p70...75. | 1E+00 |
|  |  |  |  |  |  | C/A | Hu.MIP.1a..55. | 1 | D/C | Hu.IL.12.p70...75. | 1E+00 |
|  |  |  |  |  |  | C/B | Hu.MIP.1a..55. | 1 | D/E | Hu.IL.13..51. | 1E+00 |
|  |  |  |  |  |  | C/A | Hu.MIP.1b..18. | 1 | D/A | Hu.IL.13..51. | 1E+00 |
|  |  |  |  |  |  | C/A | Hu.PDGF.bb..47. | 1 | D/B | Hu.IL.13..51. | 1 |
|  |  |  |  |  |  | C/B | Hu.PDGF.bb..47. | 1 | D/C | Hu.IL.13..51. | 1 |
|  |  |  |  |  |  | C/E | Hu.RANTES..37. | 1 | D/A | Hu.IL.15..73. | 1 |
|  |  |  |  |  |  | C/A | Hu.RANTES..37. | 1 | D/B | Hu.IL.15..73. | 1 |
|  |  |  |  |  |  | C/B | Hu.RANTES..37. | 1 | D/C | Hu.IL.15..73. | 1 |
|  |  |  |  |  |  | C/A | Hu.TNF.a..36. | 1 | D/A | Hu.IL.1ra..25. | 1 |
|  |  |  |  |  |  | C/B | Hu.TNF.a..36. | 1 | D/C | Hu.IL.1ra..25. | 1 |
|  |  |  |  |  |  | C/A | Hu.VEGF..45. | 1 | D/A | Hu.IL.2..38. | 1 |
|  |  |  |  |  |  | C/B | Hu.VEGF..45. | 1 | D/C | Hu.IL.2..38. | 1 |
|  |  |  |  |  |  |  |  |  | D/A | Hu.IL.4..52. | 1 |
|  |  |  |  |  |  |  |  |  | D/B | Hu.IL.4..52. | 1 |
|  |  |  |  |  |  |  |  |  | D/C | Hu.IL.4..52. | 1 |
|  |  |  |  |  |  |  |  |  | D/A | Hu.IL.5..33. | 1 |
|  |  |  |  |  |  |  |  |  | D/C | Hu.IL.5..33. | 1 |
|  |  |  |  |  |  |  |  |  | D/A | Hu.IL.6..19. | 1 |
|  |  |  |  |  |  |  |  |  | D/B | Hu.IL.6..19. | 1 |
|  |  |  |  |  |  |  |  |  | D/C | Hu.IL.6..19. | 1 |
|  |  |  |  |  |  |  |  |  | D/A | Hu.IL.7..74. | 1 |
|  |  |  |  |  |  |  |  |  | D/A | Hu.IL.8..54. | 1 |
|  |  |  |  |  |  |  |  |  | D/B | Hu.IL.8..54. | 1 |
|  |  |  |  |  |  |  |  |  | D/C | Hu.IL.8..54. | 1 |
|  |  |  |  |  |  |  |  |  | D/C | Hu.IL.9..77. | 1 |
|  |  |  |  |  |  |  |  |  | D/E | Hu.IP.10..48. | 1 |
|  |  |  |  |  |  |  |  |  | D/B | Hu.IP.10..48. | 1 |
|  |  |  |  |  |  |  |  |  | D/C | Hu.IP.10..48. | 1 |
|  |  |  |  |  |  |  |  |  | D/A | Hu.MCP.1.MCAF...53. | 1 |
|  |  |  |  |  |  |  |  |  | D/B | Hu.MCP.1.MCAF...53. | 1 |
|  |  |  |  |  |  |  |  |  | D/A | Hu.MIP.1b..18. | 1 |
|  |  |  |  |  |  |  |  |  | D/C | Hu.MIP.1b..18. | 1 |
|  |  |  |  |  |  |  |  |  | D/E | Hu.RANTES..37. | 1 |
|  |  |  |  |  |  |  |  |  | D/A | Hu.RANTES..37. | 1 |
|  |  |  |  |  |  |  |  |  | D/B | Hu.RANTES..37. | 1 |
|  |  |  |  |  |  |  |  |  | D/C | Hu.RANTES..37. | 1 |
|  |  |  |  |  |  |  |  |  | D/A | Hu.VEGF..45. | 1 |
|  |  |  |  |  |  |  |  |  | D/B | Hu.VEGF..45. | 1 |
|  |  |  |  |  |  |  |  |  | D/C | Hu.VEGF..45. | 1 |
|  |  |  |  |  |  |  |  |  | D/C | Hu.VEGF..45. | 1 |

| **Table S6: Tukey honest significant differences post-hoc test for Bio-plex assay [Group- specific analysis]** | | | | | | |
| --- | --- | --- | --- | --- | --- | --- |
| Comparison | | Protein | Difference | Lower CI | Upper CI | Q-value |
| Malignant | Healthy | Hu.IL.8..54. | 0.95 | 0.27 | 1.64 | 0 |
| Malignant | Healthy | Hu.PDGF.bb..47. | 0.95 | 0.26 | 1.63 | 0 |
| Benign | Healthy | Hu.IL.8..54. | 0.94 | 0.25 | 1.63 | 0 |
| Malignant | Healthy | Hu.IL.4..52. | 0.85 | 0.16 | 1.53 | 0 |
| Malignant | Healthy | Hu.MIP.1b..18. | 0.82 | 0.13 | 1.51 | 0 |
| Benign | Healthy | Hu.MCP.1.MCAF...53. | 0.82 | 0.13 | 1.5 | 0 |
| Malignant | Healthy | Hu.MCP.1.MCAF...53. | 0.81 | 0.12 | 1.5 | 0 |
| Benign | Healthy | Hu.IL.4..52. | 0.77 | 0.08 | 1.46 | 0.01 |
| Malignant | Healthy | Hu.IFN.g..21. | 0.71 | 0.03 | 1.4 | 0.03 |
| Malignant | Healthy | Hu.IL.9..77. | 0.71 | 0.02 | 1.4 | 0.03 |
| Malignant | Healthy | Hu.G.CSF..57. | 0.71 | 0.02 | 1.39 | 0.03 |
| Malignant | Healthy | Hu.IL.6..19. | 0.69 | 0.01 | 1.38 | 0.04 |
| Benign | Healthy | Hu.G.CSF..57. | 0.69 | 0 | 1.37 | 0.05 |
| Benign | Healthy | Hu.IL.6..19. | 0.65 | -0.04 | 1.34 | 0.11 |
| Malignant | Healthy | Hu.IL.5..33. | 0.63 | -0.06 | 1.31 | 0.17 |
| Malignant | Healthy | Hu.IL.1b..39. | 0.61 | -0.07 | 1.3 | 0.21 |
| Malignant | Healthy | Hu.VEGF..45. | 0.61 | -0.07 | 1.3 | 0.22 |
| Malignant | Healthy | Hu.IL.7..74. | 0.61 | -0.08 | 1.3 | 0.23 |
| Malignant | Healthy | Hu.IL.17..76. | 0.61 | -0.08 | 1.29 | 0.23 |
| Malignant | Healthy | Hu.IL.15..73. | 0.6 | -0.08 | 1.29 | 0.25 |
| Malignant | Healthy | Hu.IL.2..38. | 0.59 | -0.1 | 1.27 | 0.33 |
| Malignant | Benign | Hu.PDGF.bb..47. | 0.47 | -0.09 | 1.03 | 0.37 |
| Malignant | Healthy | Hu.TNF.a..36. | 0.57 | -0.11 | 1.26 | 0.39 |

| **Table S7: Spearman Correlation between Proseek and Bio-plex assay (with p and q-values)** | | | |
| --- | --- | --- | --- |
| **Target Protein** | **Correlation** | **p.value** | **q.value** |
| PDGF.subunit.B | 0.87 | 0.00 | 0.00 |
| IL.8 | 0.45 | 0.00 | 0.00 |
| MCP.1 | 0.46 | 0.00 | 0.00 |
| IL.6 | 0.39 | 0.00 | 0.00 |
| IL.7 | 0.31 | 0.01 | 0.02 |
| VEGF.A | 0.28 | 0.02 | 0.04 |
| IFN.gamma | 0.25 | 0.03 | 0.05 |
| IL.2 | 0.21 | 0.08 | 0.12 |
| GM.CSF | 0.18 | 0.13 | 0.19 |
| IL.4 | 0.13 | 0.28 | 0.37 |
| IL.1ra | 0.11 | 0.34 | 0.41 |
| IL.12 | 0.10 | 0.38 | 0.41 |
| TNF | 0.04 | 0.72 | 0.72 |

| **Table S8: Clinical Details of CRC patients** | | | | |
| --- | --- | --- | --- | --- |
| **Dukes' stage**  **(n=)** | **A**  **(15)** | **B**  **(15)** | **C**  **(15)** | **D**  **(15)** |
| **Age** |  |  |  |  |
| Median + SD | 65 + 7.2 | 70 + 7.9 | 65 + 9.0 | 62 + 8.0 |
| **Sex** |  |  |  |  |
| Male | 66.7% | 53.3% | 46.7% | 60% |
| Female | 22.2% | 46.7% | 53.3% | 40% |
| **Location of tumor/cancer** |  |  |  |  |
| Sigmoid | 6 (40%) | 6 (40%) | 11 | 8 (53.3%) |
| Low Rectal | 2 (13.3%) | 0 | 1 (6.7%) | 1 (6.7%) |
| Caecal | 2 (13.3%) | 3 (20%) | 3 (20%) | 3 (20%) |
| Ascending colon | 2 (13.3%) | 2 (13.3%) | 0 | 1 (6.7%) |
| Transverse colon | 2 (13.3%) | 4 (26.7%) | 0 | 0 |
| Descending colon | 0 | 0 | 0 | 2 (13.3%) |
| Adenoma | 1 (6.7%) | 0 | 0 | 0 |
| **Metastasis/Location** |  |  |  |  |
| Lymph Node | 0 | 0 | 15 | 0 |
| Liver | 0 | 0 | 0 | 8 |
| Gall Bladder/Lung | 0 | 0 | 0 | 1 |
| Ovary | 0 | 0 | 0 | 1 |
| Other Colonic Regions | 0 | 0 | 0 | 5 |
